# Eucalyptus-derived essential oils alleviate microbes and modulate inflammation by suppressing superoxide and elastase release

**DOI:** 10.3389/fphar.2023.1218315

**Published:** 2023-11-21

**Authors:** Shaimaa Fayez, Mariam I. Gamal El-Din, Saad A. Moghannem, Faizul Azam, Mohamed El-Shazly, Michal Korinek, Yu-Li Chen, Tsong-Long Hwang, Nouran M. Fahmy

**Affiliations:** ^1^ Department of Pharmacognosy, Faculty of Pharmacy, Ain-Shams University, Cairo, Egypt; ^2^ Department of Botany and Microbiology, Faculty of Science, Al-Azhar University, Cairo, Egypt; ^3^ Department of Pharmaceutical Chemistry and Pharmacognosy, Unaizah College of Pharmacy, Qassim University, Unaizah, Saudi Arabia; ^4^ Graduate Institute of Natural Products, College of Pharmacy, Kaohsiung Medical University, Kaohsiung, Taiwan; ^5^ Graduate Institute of Natural Products, College of Medicine, Chang Gung University, Taoyuan, Taiwan; ^6^ Research Center for Chinese Herbal Medicine, College of Human Ecology, Chang Gung University of Science and Technology, Taoyuan, Taiwan; ^7^ Graduate Institute of Health Industry Technology, College of Human Ecology, Chang Gung University of Science and Technology, Taoyuan, Taiwan; ^8^ Department of Anesthesiology, Chang Gung Memorial Hospital, Taoyuan, Taiwan; ^9^ Department of Chemical Engineering, Ming Chi University of Technology, New Taipei City, Taiwan

**Keywords:** Eucalyptus oil, elastase, antiviral, antifungal, antibacterial, molecular docking, *in silico*

## Abstract

The Eucalyptus tree, belonging to the myrtle family, grows all over the world for its pharmaceutical and industrial benefits. In this article, we present a comparative analysis of the chemical composition of the hydrodistilled oils obtained from three different Eucalyptus species growing in Egypt viz. *E. citriodora*, *E. camaldulensis*, and *E. ficifolia*. Gas Chromatography-Mass Spectrometric guided analysis resulted in the identification of a total of 20 metabolites in *E. citriodora* oil with citronellal (54.9%) and citronellol (25.4%) being the most dominant components. *β*-cymene (12.7%) and 1,8-cineole (11.7%) were the major volatile constituents identified in *E. camaldulensis* oil, while *trans*-*β*-ocimene (22.4%), 1,8-cineole (13.5%), and *L*-*trans*-pinocarveol (12.5%) were the dominating components in the oil of *E. ficifolia*. The essential oils of the studied species were evaluated for their *in vitro* anti-inflammatory, antiviral including anti-SARS-CoV-2 (severe acute respiratory syndrome corona virus 2), antibacterial, and antifungal activities. *E. citriodora* oil displayed the highest inhibitory activity on the release of the superoxide radical (32%) and elastase enzyme (31%) in human neutrophils, while *E. ficifolia* oil had enhancing effects on elastase. The latter showed significant antiviral effects against hepatitis A, herpes simplex, and coxsackie viruses with IC_50_ values at 2.1, 2.5, and 5.6 μg/mL, respectively. Moderate antibacterial and antifungal activities were observed for Eucalyptus oils with *Staphylococcus aureus* being the most susceptible bacterial strain. *E. ficifolia* oil, similarly, displayed the best antibacterial activity with minimum inhibitory concentration (MIC) value at ca. 25 μg/mL (for *S. aureus*). On the contrary, *E. camaldulensis* oil was the most active against *Candida albicans* with an MIC value at 45 μg/mL. *In silico* studies were performed with a number of macromolecular drug targets for confirming the biological activities of the identified compounds and for interpreting their ADME (absorption-distribution-metabolism-elimination) parameters.

## 1 Introduction

The Eucalyptus L’ Her, known as the gum tree (family Myrtaceae), is cultivated worldwide for its medicinal, aromatic, and industrial purposes. Genus Eucalyptus, native to Australia, harbors over 800 species of tall, evergreen, woody perennials trees. Traditionally, Eucalyptus species were used for the management of fever, cold, and flu symptoms (Silva et al., 2003b). The different species of Eucalyptus have long been used traditionally for their analgesic, and anti-inflammatory effects and for managing cold and respiratory infections (Silva et al., 2003a). Previous studies reported the significant inhibitory effects of Eucalyptus oils on resistant Gram (+) and Gram (−) bacterial strains, nematodes, fungi, and insects (Sugumar et al., 2014; Alipanah et al., 2022; Elangovan and Mudgil, 2023; Ez-Zriouli et al., 2023). Antihistaminic, antidiabetic, analgesic, and wound healing potentials were likewise reported on Eucalyptus essential oils (Sartorelli et al., 2007; El-Shiekh et al., 2020). On the industrial scale, the bark showed strong chromium (VI) absorbent power which could be beneficial in the removal of chromium from industrial waste (Sarin and Pant, 2006). Chemical investigations on *Eucalyptu*s essential oils revealed that they are a rich source of monoterpenes, sesquiterpenes, and oxygenated terpene derivatives including esters, ketones, alcohols, phenols, and aldehydes, that contributed to the utility of Eucalyptus oils in food, perfumery, and pharmaceutical industries (Salehi et al., 2019).

Inflammation and microbial resistance are two major health issues that pose huge challenges on the healthcare system. Inflammation is a key player in the rapid progress of diseases with high mortality rate including cancer, autoimmune disorders, cardiovascular problems, obesity and diabetes, while microbial resistance resulted in the inefficacy of the current in use antibiotics, antivirals, antifungals, and anti-parasites in combating human pathogens (Wang et al., 2019). Human neutrophils are cornerstones in the body defense against xenobiotics, microorganisms, and the initiation of inflammatory reactions through the release of superoxide radicals (O_2_^•-^), which are important biomarkers of inflammation (Ley et al., 2018).

Elastase is a serine protease immunomodulatory enzyme that plays a crucial role in inflammation (Crocetti et al., 2019) and in the management of bacterial infections (Khomtchouk et al., 2021). Its inhibition resulted in positive outcomes in patients with inflammatory disorders like chronic kidney diseases (CKD) and chronic obstructive pulmonary disease (COPD) (Bronze-Da-Rocha and Santos-Silva, 2018). The rapid progress in the discovery of new antivirals by the pharmaceutical sector is attributed to the sharp rise in viral-associated diseases. According to the CDC (centre for disease control and prevention), several viral infections are widespread. Hepatitis A infections increased dramatically during 2016–2018 with an occurrence rate of 3.8 cases/100,000 population (Monique et al., 2021). Herpes simplex 1 affected ca. 3.7 billion people globally (Kuny et al., 2020) with symptoms ranging from mild uncomplicated skin infection to life-threatening encephalitis (Whitley and Roizman, 2001). Coxsackie B viruses are involved in a variety of human diseases like cardiomyopathy, hepatitis, and diabetes type 1 (Akuzawa et al., 2014).

*Candida albicans* despite being part of the gut flora yet constitutes the most prevailing human fungal pathogen causing infections that could be life-threatening especially in immunocompromised patients. Invasive candidiasis can seriously affect the heart, blood, brain, bones, and other body organs (Berman, 2012). Failure in rapid treatment of these hazardous microbial infections often results in serious complications and even death (Ünüvar, 2018). Therefore, there is an uprising need for the discovery of antimicrobial alternatives to reduce the incidence of resistance.

Despite the great diversity of species within *Eucalyptus*, only few of them were studied for their essential oil chemistry and pharmacology. Besides, reports addressing the effect of Eucalyptus oil on neutrophil elastases are scanty. Herein, we conducted a GC-MS-assisted comparative analysis of the chemical profiles of the essential oils obtained from three Eucalyptus species growing in Egypt, viz. *E. citriodora* Hook., *E. ficifolia* F.Muell., and *E. camaldulensis* Dehnh. The oils were further investigated for their potential inflammatory-modulating effects, antibacterial, antiviral, and antifungal activities. The analysis was supported by *in silico* studies of the major identified constituents on protein targets linked to human neutrophil elastase, *S. aureus*, hepatitis A virus, *Herpes simplex* 1, and *Candida albicans* to justify the biological activities of the studied oils.

## 2 Materials and methods

### 2.1 Plant material

An amount of 1 kg of the fresh leaves of *E. citriodora*, *E. camaldulensis*, and *E. ficifolia* were collected in summer 2020 from the trees growing in the Egyptian zoo in Giza. Plant authentication was done by the agricultural consultant, Therease Labib at Mazhar botanical garden. Voucher specimens were accessioned at Pharmacognosy department herbarium, Faculty of Pharmacy, Ain Shams University, Cairo, Egypt (Codes: PHG-P-EC-384, PHG-P-EC385, and PHG-P-EF-386 for *E. citriodora*, *E. camaldulensis*, and *E. ficifolia*, respectively). The plant names were checked with http://www.theplantlist.org/tpl1.1/search?q=Eucalyptus.

### 2.2 Preparation of Eucalyptus essential oils

Two hundred grams of *E. citriodora*, *E. camaldulensis*, and *E. ficifolia* fresh leaves were hydrodistilled for ca. 4 h using a Clevenger system to extract the essential oils. Residual moisture was removed by drying over anhydrous sodium sulphate then the essential oils were stored in amber-sealed vials at −20°C. The oil yield was calculated as %w/w relative to the initial plant weight.

### 2.3 Chemical analysis of essential oils using GC-MS

Essential oils analysis was performed on gas chromatography coupled to mass spectrometry (GC-MS) utilizing a Shimadzu GC-MS-QP 2010 system (Koyoto, Japan) following the method previously described by the authors (Fayez et al., 2022). Identification of the compounds was accomplished *via* comparison of the relative retention indices and mass spectra of the detected compounds with the data reported in Wiley database, NIST-17, and in the published literature (Senthil Kumar et al., 2020; Gamal El-Din et al., 2022).

### 2.4 Assessment of the *in vitro* anti-inflammatory activity of Eucalyptus oils

#### 2.4.1 Preparation of human neutrophils

Healthy human aged from 20 to 35 years old was volunteered for blood donation by venipuncture utilizing the protocol approved by the institutional review board at Chang Gung Memorial Hospital and administered according to the guidelines of the Declaration of Helsinki (IRB no. 201902217A3). The neutrophils were isolated and prepared using a previously reported protocol (Yang et al., 2013; Al-Sayed et al., 2020).

#### 2.4.2 Measurement of superoxide generation

The superoxide release in human neutrophils was assessed using ferricytochrome *c* (Korinek et al., 2021a; El-Din et al., 2022) based on the method described by Korinek et al. (2021b). Neutrophils (6 × 10^5^/mL) supplemented with 0.6 mg/mL ferricytochrome *c* (Sigma-Aldrich, St. Louis, MO, United States) were incubated with the tested compounds or DMSO (control) for 5 min. Cells were activated with formyl-methionyl-leucyl-phenylalanine (fMLF, 100 nM)/cytochalasin B (CB, 1 μg/mL) for 10 min. The absorbance was continuously monitored at 550 nm (Hitachi U-3010, Hitachi Inc., Tokyo, Japan). The results were calculated by measuring the variations in the absorbance values and dividing the result by the extinction coefficient of the reduced ferricytochrome *c*. Genistein served as a positive control.

#### 2.4.3 Measurement of elastase release

Elastase release was calculated according to reported procedure (Hwang et al., 2006; El-Din et al., 2022) *via* degranulation of azurophilic granules in human neutrophils. The percentage absorbance values of elastase release was demonstrated through the results in both the fMLF/CB-activated and the drug-free control system. Monitoring of absorbance changes at 405 nm was carried out using the spectrometer (HitachiU-3010, Tokyo, Japan). Genistein served as a positive control.

### 2.5 Assessment of the *in vitro* anti-microbial activity of Eucalyptus oils

#### 2.5.1 Assessment of the *in vitro* antiviral activity

The rapidly growing viral strains in VERO cells were used in the current study. Herpes Simplex Virus type 1 (HSV-1), Hepatitis A virus-H10 (HAV H10), and Coxsackie virus (COX-B4) were isolated using the protocol developed by Elsehemy et al. (2020) and Yang et al. (2022) and Fahmy et al. (2020). The quantal assay was used to assess the viral infection of Vero cells for obtaining the plaque formation unit (PFU) and the 50% tissue culture infectious dose end-point (TCID50%) (Reed and Muench, 1938) and Fahmy et al. (2020). The MTT assay was performed to test the antiviral activities of essential oils against the selected viruses in Vero cells in presence of acyclovir =9-(2-hydroxyetho-xymethyl)guanosine (Sigma-Aldrich), a standard antiviral drug, as previously described by Fahmy et al. (2020). The 50% viral inhibitory concentration (EC_50_) was calculated from the dose-response curve following linear regression analysis. In the case of SARS-CoV-2, a pseudo-typed assay was performed using stable human angiotensin-converting enzyme 2 (hACE-2) overexpressed HEK293T cells (offered by Dr. Rei-Lin Kuo from Chang Gung University) and preserved in DMEM (Dulbecco’s Modified Eagle Medium) supplied with 10% FBS (fetal bovine serum) and 10 μg/mL blasticidin. The VSV-G (vesicular stomatitis virus G protein) pseudotyped lentivirus control (clone name: S3w.Fluc. Ppuro) and SARS-CoV-2 spike protein expressing VSV-G pseudo-typed lentiviruses (clone name: nCoV-S-Luc-D614G and nCoV-S-Luc-B.1.617.2) were purchased from RNAi Core Facility of Academia Sinica. The hACE-2 overexpressed cells (in a concentration of 1 × 104 cells/well) were seeded on 96 well plates and incubated at 37°C, 5% CO2. Equal relative infection unit (RIU) (5 × 103 RIU/well = 0.5 RIU/cell) of pseudo-typed lentiviruses were pretreated with different concentrations of the tested oils or DMSO (dimethyl sulfoxide) in DMEM containing 5% FBS at 37°C for 1 h. The medium of ACE-2 (angiotensin converting enzyme 2) overexpressed cells was replaced with treated pseudo-typed lentivirus and cultured for 24 h. Viral infection was determined according to the luciferase activity measured by a luciferase assay system kit (Promega, WIS, United States) and recorded using a Tecan Infinite F200 Pro fluorescence multiplate reader (Männedorf, Switzerland).

#### 2.5.2 Assessment of the *in vitro* antibacterial activity

The antibacterial activity assay of the oils was accomplished using the following bacterial strains, *Klebsiella pneumonia* ATTC 700603*, Enterococcus faecalis* ATCC 29212*, Salmonella typhi* ATTC 6539*, Escherichia coli* ATTC 25922, and *Staphylococcus aureus* ATTC 25923. The Kirby-Bauer disc diffusion method was adopted and the minimum inhibitory concentration (MIC) was assessed following a previously described methodology (Perez et al., 1990; El-Sherbiny et al., 2023). Chloramphenicol, 2,2-Dichloro-N-[(1R,2R)-1,3-dihydroxy-1-(4-nitrophenyl)-2-propanyl]acetamide (Sigma-Aldrich) was used as the standard drug.

#### 2.5.3 Assessment of the *in vitro* antifungal activity

A clinical isolate *Candida albicans* was obtained from the regional centre of Mycology and Biotechnology, Al-Azhar University, Cairo, Egypt. The assay was performed using the disc diffusion method as previously described. Fluconazole (Bioanalysis, Ankara, Turkey) was used as a standard antifungal drug.

### 2.6 Statistical analysis

Results were expressed as mean ± S.E.M of at least three independent measurements. The Student’s t-test was employed for the performed statistical analysis (Systat Software Inc., Sigma Plot, Systat Software, San Jose, CA, United States). Values with *p < 0.05, **p < 0.01 and ***p < 0.001 were considered statistically significant.

### 2.7 Molecular docking studies

The PubChem database (https://pubchem.ncbi.nlm.nih.gov/) was used to retrieve the three-dimensional structures of the ligands used for molecular docking in this study. The proteins’ X-ray structural coordinates were downloaded from the protein data bank (https://www.rcsb.org/). Initial processing of the receptor and ligand files was performed in AutoDock Tools 1.5.7 and Biovia Discovery Studio Visualizer 2021. In AutoDock Tools 1.5.7, each ligand in PDB format was handled to merge all non-polar hydrogens, assign partial charges, and define rotatable bonds and a torsion tree. Molecular docking was accomplished using the AutoDock Vina 1.1.2 program, keeping the exhaustiveness value of 100 and the default software settings for the rest of the parameters (Trott and Olson, 2010). At the centre of each co-crystallized ligand, a grid box with dimensions of 24, 24, and 24 points in the x, y, and z directions was constructed in the receptor. After docking computation, the Biovia Discovery Studio Visualizer 2021 and PyMol programs were used to analyse the best configurations of each ligand in terms of their binding energy (*G*_binding_, kcal/mol) and molecular interactions (Fahmy et al., 2020; Azam, 2021).

## 3 Results

### 3.1 Extraction and distillation of essential oils

Hydro-distillation of the fresh leaves of the different Eucalyptus species yielded clear dark yellow oils lighter than water. The yields were expressed as the weight of the oil per 100 g fresh leaves and constituted 0.74%, 0.37%, and 0.45% on dry weight basis for *E. citriodora, E. camaldulensis*, and *E. ficifolia*, respectively.

### 3.2 GC-MS-guided profiling of the chemical composition of Eucalyptus essential oils

**FIGURE 1:**
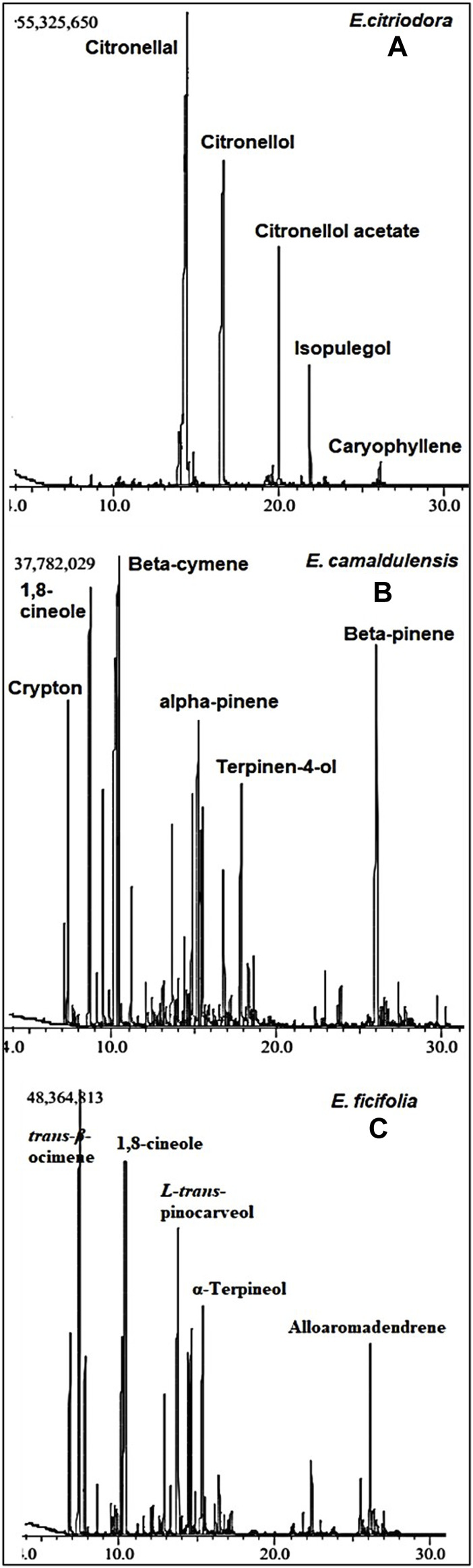
The GC-MS chromatograms of *E. citriodora* **(A)**, *E. camaldulensis* **(B)**, and *E. ficifolia* **(C)** oils collected from Giza, Egypt.

**FIGURE 2:**
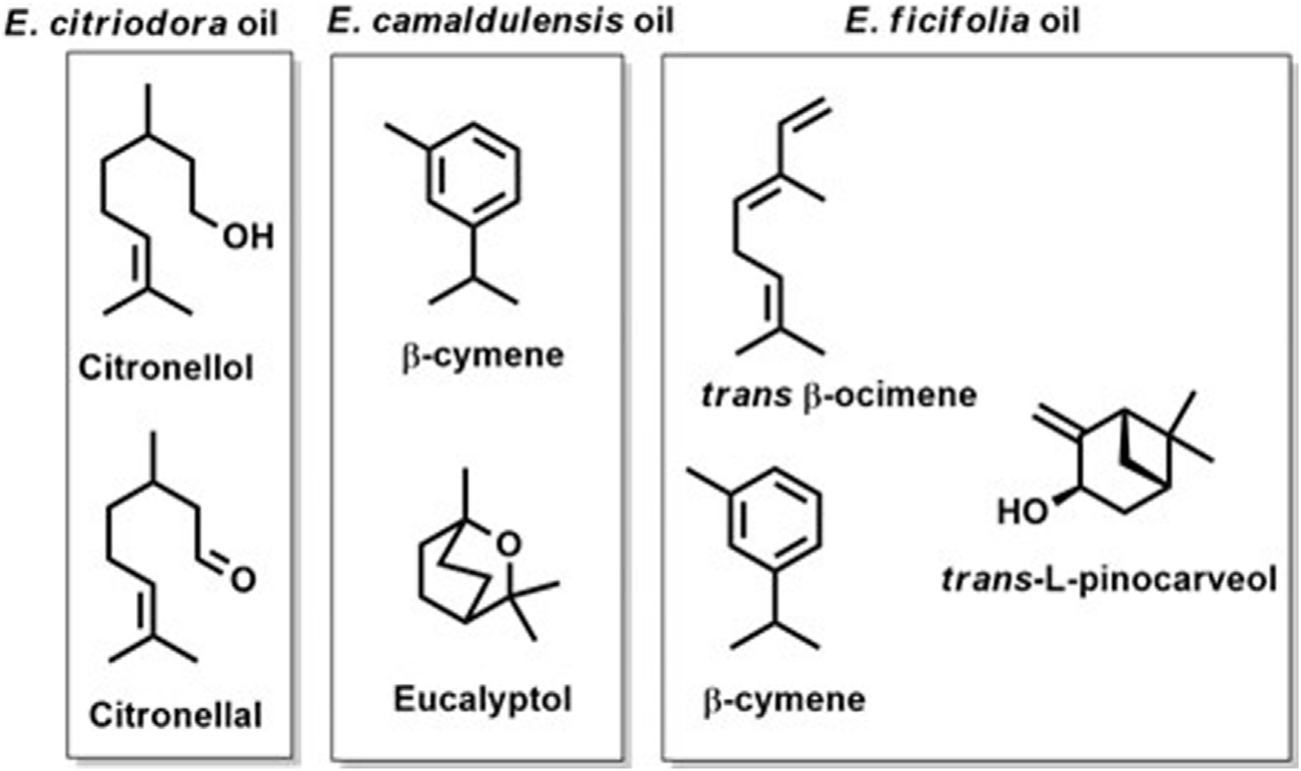
The structures of the major volatile components identified in the oil of *E. citriodora*, *E. camaldulensis*, and *E. ficifolia*.

The GC-MS chromatograms of the obtained oils hinted at the presence of monoterpenes, sesquiterpenes, phenols, oxides, alcohols, esters, aldehydes, and ketones. The identified metabolites were arranged based on their order of elution from the DB-5 column. A total of twenty volatile components were identified in *E. citriodora* oil representing ca. 96.98% of its total oil content (Table 1). The monoterpenoid aldehyde citronellal, was found to be the major metabolite constituting more than 50% of the oil (54.97%). The acyclic monoterpenoidal alcohol, citronellol, and its acetate derivative accounted for 25.42% and 7.37% of citriodora oil, respectively, while the monoterpene alcohol, isopulegol, and the sesquiterpene hydrocarbon, caryophyllene, displayed 4.34% and 2.87% of *E. citriodora*, respectively. Meanwhile, forty volatile constituents were identified from the essential oil of *E. camaldulensis*, belonging to the monoterpene hydrocarbons and their oxygenated derivatives (Figure 1B). Major constituents were β-cymene, 1,8-cineole, β-pinene, crypton, α-pinene, and terpinen-4-ol, accounting for 12.77%, 11.74%, 9.86%, 7.2%, 4.1%, and 3.5%, respectively. In the essential oil of *E. ficifolia,* thirty-nine volatile constituents were identified (Figure 1C), constituting ca. 89.1% of the total hydrodistilled oil content. The major dominating terpenes were *trans*-β-ocimene (22.4%), 1,8-cineole (13.5%), and L-*trans*-pinocarveol (12.5%) (Table 1). Figure 2 shows the structures of the major volatile components identified in the oil of *E. citriodora*, *E. camaldulensis*, and *E. ficifolia*.

**TABLE 1:** Volatile constituents identified in the essential oils of *E. camaldulensis*, *E. citriodora*, and *E. ficifolia*.

No.	Compounds^[a]^	*R*_*t*_	*RI*		MF^[d]^	Composition (%)		
			Measured^[b]^	Reported^[c]^		*E. citriodora*	*E. camaldulensis*	*E. ficifolia*
1	Propanoic acid	6.821	897	766	C_8_H_16_O_2_	-	-	2.66
2	α-Thujene	7.135	908	906	C_10_H_16_	-	0.91	-
3	α-Pinene	7.360	915	916	C_10_H_16_	0.15	4.16	0.01
4	*trans*-*β*-Ocimene	7.501	922	1,050	C_10_H_16_	-	-	22.40
5	Camphene	7.745	931	932	C_10_H_16_	-	0.15	2.47
6	2,4(10)-Thujadiene	7.920	937	945	C_10_H_14_	-	0.09	-
7	*β*-Pinene	8.685	965	964	C_10_H_16_	0.19	9.86	0.58
8	*β*-Myrcene	9.065	979	978	C_10_H_16_	0.06	0.59	-
9	2-Carene	9.330	988	992	C_10_H_16_	-	0.04	-
10	α-Phellandrene	9.470	993	995	C_10_H_16_	-	2.61	0.42
11	Isobutyl isovalerate	9.537	996	1,003	C_9_H_18_O_2_	-	-	0.30
12	Propanoic acid, 2-methyl-, 3-methylbutyl ester	9.743	1,003	1,020	C_9_H_18_O_2_	-	-	0.54
13	α-Terpinene	9.835	1,006	1,007	C_10_H_16_	-	0.50	-
14	Propanoic acid, 2-methyl-, 2-methylbutyl ester	9.863	1,007	1,014	C_9_H_18_O_2_	-	-	0.65
15	*β*-Cymene	10.240	1,019	1,019	C_10_H_14_	0.07	12.77	6.21
16	1,8-Cineole (Eucalyptol)	10.405	1,024	1,024	C_10_H_18_O	-	11.74	13.51
17	*β*-Ocimene	10.535	1,028	1,027	C_10_H_16_	0.09	0.46	-
18	*γ*-Terpinene	11.165	1,049	1,049	C_10_H_16_	0.13	2.06	0.17
19	Camphinelone	11.930	1,073	1,082	C_10_H_14_O	-	0.03	-
20	*p*-Mentha-2,4(8)-diene	12.071	1,078	1,079	C_10_H_16_	-	0.70	0.39
21	Isovaleric acid, isopentyl ester	12.597	1,095	1,094	C_10_H_20_O_2_	-	-	0.30
22	Isovaleric acid, 2-methylbutyl ester	12.689	1,098	1,107	C_10_H_20_O_2_	-	-	0.21
23	Fenchol	12.875	1,104	1,110	C_10_H_18_O	-	0.29	2.65
24	Thujone	12.945	1,106	1,102	C_10_H_16_O	-	0.18	-
25	α-Campholenal	13.245	1,116	1,111	C_10_H_16_O	-	0.30	0.71
26	2(10)-Pinen-3-ol	13.690	1,130	1,138	C_10_H_16_O	-	3.08	-
27	L-*trans*-Pinocarveol	13.815	1,134	1,137	C_10_H_16_O	-		12.55
28	4(10)-Thujen-3-ol	13.875	1,136	1,137	C_10_H_16_O	-	0.37	-
29	Isopulegol	13.897	1,136	1,145	C_10_H_18_O	4.34	-	0.25
30	(*R*)-(+)-Citronellal	14.127	1,144	1,148	C_10_H_18_O	54.97	-	0.11
31	Pinocarvone	14.400	1,153	1,155	C_10_H_14_O	-	0.91	3.49
32	L-Borneol	14.565	1,158	1,159	C_10_H_18_O	-	0.51	4.77
33	Terpinen-4-ol	14.895	1,169	1,171	C_10_H_18_O	-	3.54	-
34	Crypton	15.225	1,179	1,180	C_9_H_14_O	-	7.26	-
35	α-Terpineol	15.345	1,183	1,183	C_10_H_18_O	0.06	2.27	6.91
36	Myrtenol	15.500	1,188	1,191	C_10_H_16_O	-	2.66	0.70
37	2-Pinen-4-one	15.860	1,200	1,202	C_10_H_14_O	-	0.56	-
38	*cis*-Carveol	16.149	1,210	1,214	C_10_H_16_O	-	0.36	0.57
39	*cis*-*p*-Mentha-1(7),8-dien-2-ol	16.417	1,219	1,231	C_10_H_16_O	-	-	1.24
40	Citronellol	16.590	1,225	1,228	C_10_H_20_O	25.42	-	-
41	Cumaldehyde	16.780	1,232	1,230	C_10_H_20_O	-	1.98	-
42	Pulegone	16.795	1,232	1,233	C_10_H_16_O	0.01	-	-
43	D-Carvone	16.895	1,236	1,234	C_10_H_14_O	-	0.21	0.09
44	Carvotanacetone	16.986	1,239	1,246	C_10_H_16_O	-	-	0.33
45	Piperitone	17.185	1,246	1,248	C_10_H_16_O	-	0.55	0.48
46	Isopulegol acetate	17.775	1,267	1,267	C_12_H_20_O_2_	-		
47	Phellandral	17.830	1,269	1,272	C_10_H_16_O	-	3.49	-
48	*p*-Cymen-7-ol	18.275	1,284	1,286	C_10_H_14_O	-	0.90	-
49	*p-*Mentha-1,8-dien-7-ol	18.500	1,292	1,295	C_10_H_16_O	-	0.29	-
50	Carvacrol	18.585	1,295	1,295	C_10_H_14_O	-	1.44	0.18
51	(*R*)-(+)-Citronellic acid	19.293	1,320	1,315	C_10_H_18_O	0.83	-	
52	Citronellol acetate	19.970	1,343	1,343	C_12_H_18_O	7.37	-	0.09
53	α-Copaene	20.595	1,365	1,365	C_15_H_24_	-	0.02	-
54	Isobutyric acid, phenethyl ester	21.145	1,384	1,396	C_12_H_16_O_2_	-	-	0.22
55	Caryophyllene	21.812	1,409	1,409	C_15_H_24_	2.87	-	0.31
56	Aromandendrene	22.330	1,429	1,447	C_15_H_24_	0.11	0.28	1.22
57	Humulene	22.730	1,445	1,445	C_15_H_24_	0.21	-	0.02
58	9-epi-*trans*-Caryophyllene	22.922	1,452	1,455	C_15_H_24_	-	-	0.24
59	Alloaromadendrene	22.930	1,452	1,452	C_15_H_24_	0.04	0.62	3.88
60	(+)-Ledene	23.811	1,487	1,487	C_15_H_24_	-	-	0.15
61	(−)-Globulol	25.665	1,559	1,560	C_15_H_26_O	-	0.18	0.88
62	Spathulenol	26.050	1,574	1,574	C_15_H_24_O	-	9.05	-
63	Humulene epoxide	26.715	1,600	1,600	C_15_H_24_O	0.02	-	
64	Ƭ-Cadinol	27.459	1,633	1,637	C_15_H_26_O	-	-	0.12
65	Thunbergol	35.320	1992	2032	C_20_H_34_O	0.04	-	-
Monoterpene hydrocarbons						0.69	34.90	32.65
Oxygen containing monoterpene						93.00	42.92	39.05
Sesquiterpene hydrocarbons						3.23	0.92	1.92
Oxygen containing sesquiterpene						0.02	9.23	1
Others						0.04	-	4.37
Total identified components						96.98	87.97	89.10

### 3.3 *In vitro* anti-inflammatory activity of Eucalyptus oils

#### 3.3.1 The influence of essential oils on the release of superoxide free radicals in fMLF/CB-induced human neutrophils

At the dose of 10 μg/mL, the essential oil of *E. citriodora* displayed the highest potency on suppressing superoxide radicals by 32% in human neutrophils activated by fMLF/CB (Table 2). On the other hand, the oils of *E. camaldulensis* and *E. ficifolia* showed lower efficacy in inhibiting superoxide ions which is probably attributed to the variation in their chemical composition and the percentage of different components among the oils of the three Eucalyptus species. Genistein, a known natural tyrosine kinase inhibitor, was used as a positive control (Korinek et al., 2021a).

**TABLE 2:** Effect of *E. citriodora, E. camaldulensis*, and *E. ficifolia* essential oils on the release of superoxide free radical.

Essential oil	(%) Inhibition of superoxide anion
*E. citriodora*	32.53 ± 5.95^a^
*E. camaldulensis*	13.60 ± 3.74*
*E. ficifolia*	19.39 ± 5.78*
Genistein^b^	92.72 ± 3.20

^a^ % inhibition at 10 μg/mL. Results are presented as mean ± SEM (at *n* = 3–4). *p < 0.05, compared with the control (DMSO).

^b^ Genistein (10 µM) positive control.

#### 3.3.2 The influence of essential oils on elastase release in fMLF/CB-induced human neutrophils

The oil of *E. citriodora,* at a concentration of 10 μg/mL, inhibited the release of human neutrophil elastase by 31%, therefore reducing the degranulation triggered by fMLF/CB in the neutrophils (Table 3). Genistein inhibited 50.96 ± 8.57% of superoxide anion generation. Meanwhile, the essential oil of *E. ficifolia* was found to increase the release of elastase from human neutrophils by 85.85 ± 1.99% with the absence of fMLF or CB, demonstrating its ability to promote inflammatory responses through immunomodulatory effects which reveals that *E. ficifolia* oil might be a promising candidate for antimicrobial studies.

**TABLE 3:** Inhibitory effects of *E. citriodora, E. camaldulensis*, and *E. ficifolia* essential oils on the release of elastase enzyme.

Essential oil	(%) Inhibition of elastase release
*E. citriodora*	31.84 ± 1.64***
*E. camaldulensis*	21.30 ± 3.11**
*E. ficifolia*	Increasing^a^
Genistein^b^	50.96 ± 8.57

*% Inhibition at 10 μg/mL. Results are presented as mean ± SEM (n = 3–4). *p < 0.05, **p < 0.01, ***p < 0.001 compared with the control (DMSO).

^a^ *E. ficifolia* increased release of elastase from human neutrophils by 85.85% ± 1.99% without presence of primer (cytochalasin B, CB) or activator (fMLF).

^b^ Genistein (10 µM) positive control.

### 3.4 *In vitro* antimicrobial activity of Eucalyptus oils

#### 3.4.1 Antiviral activity

The antiviral activity of *E. citriodora, E. camaldulensis,* and *E. ficifolia* was evaluated against Hepatitis A, Herpes simplex 1, and Coxsackie B4 viruses using MTT assay in Vero cells. The maximum non-toxic concentration (MNTC) of the oils were evaluated against 100 tissue culture infectious dose TCID50/mL of viruses (Barakat et al., 2010; Zandi et al., 2010) (Table 3). Results revealed that the essential oil of *E. ficifolia* exhibited the highest antiviral activity against HAV-H10, HSV-1, and COX-B4 with IC_50_ at 2.1 ± 0.87 μg/mL, 2.57 ± 0.9 μg/mL, and 5.6 ± 1.35 μg/mL, respectively. *E. citriodora* oil showed a moderate effect against HSV-1 and COX-B4, but no activity observed against HAV-H10. Meanwhile the three essential oils demonstrated insignificant activities against SARS-Cov-2 pseudo-typed infection at 50 μg/mL (data not shown).

#### 3.4.2 Antibacterial activity

Assessment of the antibacterial activities of the essential oils was performed against both Gram-positive and Gram-negative bacteria using the disc diffusion method. Results revealed that *E. citriodora, E. camaldulensis*, and *E. ficifolia* oils at the concentration of 1 mg/mL were active against all tested strains, except for *E. citriodora* oil which was inactive against the Gram-negative bacteria like *E. coli* and *S. typhi*. The Gram-positive *S. aureus* showed the highest sensitivity against *E. citriodora, E. ficifolia*, and *E. camaldulensis* oils with inhibition zone diameters of 32 ± 1.3 mm, 34 ± 0.8 mm, and 36 ± 1.0 mm, respectively, compared with 25 ± 1.6 mm for the standard chloramphenicol (Table 4). The minimum inhibitory concentrations (MICs) of the different Eucalyptus oils are recorded in Table 5 where *E. ficifolia* oil displayed the best activity against all bacterial strains.

**TABLE 4:** The antiviral activity of *E. citriodora*, *E. camaldulensis,* and *E. ficifolia* essential oils.

Tested sample	CC_50_ ^a^ (µg/mL) ±SD	IC_50_ ^b^ (µg/mL) ±SD^c^		
		HAV-H10	HSV-1	COX-B4
*E. citriodora*	70.2 ± 2.1	NA	2.63 ± 1.61	8.9 ± 0.67
*E. camaldulensis*	108.7 ± 4.1	13.6 ± 0.56	15.6 ± 1.2	25.4 ± 0.32
*E. ficifolia*	85.3 ± 3.4	2.1 ± 0.87	2.57 ± 0.9	5.6 ± 1.35
Acyclovir	7.1 ± 0.22	NA^d^	0.62 ± 0.34	NA

^a^ Toxic concentration to 50% of the Vero cells (CC_50_).

^b^ Concentration of the drug which causes 50% of viral inhibition (IC_50_).

^c^ Standard deviation (SD).

^d^ Not active at MNTC (mean non-toxic concentration).

**TABLE 5:** Antibacterial and antifungal (expressed as inhibition zone diameter) activities of *E. citriodora*, *E. camaldulensis*, and *E. ficifolia* essential oils.

Tested strain	Inhibition zone diameter (mm)			Standard drug
	*E. citriodora*	*E. camaldulensis*	*E. ficifolia*	
Bacteria				Chloramphenicol (30 μg/mL)
*K. pneumonia*	17 ± 1.1	23 ± 2.0	19 ± 1.5	20 ± 0.6
*E. faecalis*	13 ± 0.9	18 ± 1.7	17 ± 1.9	22 ± 2.2
*E. coli*	NA	13 ± 0.6	16 ± 2.1	11 ± 1.2
*S. typhi*	NA	15 ± 1.1	13 ± 0.6	31 ± 0.3
*S. aureus*	32 ± 1.3	36 ± 1.0	34 ± 0.8	25 ± 1.6
Fungi				Fluconazole (25 μg/mL)
*C. albicans*	20 ± 0.9	25 ± 1.3	23 ± 1.3	18 ± 0.5

NA: not active at 1 mg/mL.

#### 3.4.3 Antifungal activity

The antifungal activities of the three essential oils were assessed against the opportunistic pathogenic fungus, *Candida albicans* (Table 5; Table 6). The results showed that *E. camaldulensis* oil displayed the highest potency followed by *E. citriodora* and *E. ficifolia* with MIC values at 45.3 ± 1.7, 65.1 ± 1.6, and 71.1 ± 3.6 μg/mL, respectively, compared with the standard fluconazole (14.5 ± 3.2 μg/mL). The potent antifungal activity displayed by *E. camaldulensis* essential oil is probably attributed to its major constituents, β-cymene and β-pinene, previously reported for their fungistatic activities (Tampieri et al., 2005; Höferl et al., 2009).

**TABLE 6:** The minimum inhibitory concentrations (MICs) of *E. citriodora, E. camaldulensis*, and *E. ficifolia* essential oils against the tested bacteria and fungi.

Tested strain	MIC (µg/mL)			Standard drug
	*E. citriodora*	*E. camaldulensis*	*E. ficifolia*	
Bacteria				Chloramphenicol
*K. pneumonia*	53.2 ± 1.2	71.6 ± 1.6	51.3 ± 2.4	6.9 ± 0.43
*E. faecalis*	45.4 ± 2.31	55.4 ± 4.2	40.8 ± 5.1	8.1 ± 1.3
*E. coli*	ND	74.2 ± 3.9	72.4 ± 2.8	12.9 ± 0.22
*S. typhi*	ND	85.3 ± 5.2	65.2 ± 1.9	11.3 ± 1.6
*S. aureus*	42.6 ± 3.6	27.8 ± 2.3	25.6 ± 4.6	2.1 ± 0.5
Fungi				Fluconazole
*C. albicans*	65.1 ± 1.6	45.3 ± 1.7	71.1 ± 3.6	14.5 ± 3.2

ND: not determined.

### 3.5 Molecular docking studies

The molecular interactions between small compounds and the receptor binding cavity can be accurately predicted by molecular docking. To gain a theoretical understanding of the biological activity of the major oil constituents of Eucalyptus species, 1-8-cineole, β-cymene, citronellal, citronellol, *trans*-β-ocimene, and L-*trans*-pinocarveol, molecular docking experiment was performed using therapeutic targets linked to *Candida albicans*, human neutrophil elastase, *Herpes simplex* virus type-1, hepatitis A virus, and *Staphylococcus aureus*. The typical structural framework of the compounds under investigation allows them to interact with a wide range of pharmacological targets which help to explain the observed biological activity. Table 7 shows the docking-predicted binding energy, whereas Supplementary Figures S1–S13 in the Supporting Material exhibit an elaborated intermolecular interaction of the minimum energy conformation of each docked molecule.

**TABLE 7:** Molecular docking score of major identified compounds in terms of binding energy (kcal/mol).

Targets/Compounds		1,8-Cineole	*β*-Cymene	Citronellal	Citronellol	*trans*-*β*-Ocimene	L-*trans*-Pinocarveol
*Candida albicans*	CYP51	−6.7	−6.0	−6.8	−6.0	−6.4	−6.8
	Dihydrofolate reductase	−4.8	−4.8	−5.9	−4.8	−5.2	−4.9
	N-myristoyltransferase	−5.9	−5.1	−6.0	−5.5	−5.6	−6.1
	Secreted aspartic proteinase	−5.5	−4.9	−5.6	−5.0	−5.0	−5.5
	Thymidylate kinase	−5.6	−6.4	−7.7	−6.6	−7.0	−5.8
Human neutrophil elastase	Elastase	−4.9	−4.2	−5.3	−4.5	−4.5	−5.4
Hepatitis A virus	3C proteinase	−4.4	−3.7	−4.4	−3.9	−4.0	−4.4
*Herpes simplex* virus type-1	DNA polymerase	−4.8	−4.3	−5.5	−4.5	−4.4	−5.1
	Thymidine kinase	−5.9	−5.9	−7.3	−6.0	−6.4	−6.2
*Staphylococcus aureus*	Dihydrofolate reductase	−5.8	−5.1	−5.7	−5.3	−5.4	−5.8
	DNA gyrase	−4.9	−5.1	−5.8	−5.6	−5.6	−5.2
	Pantothenate synthetase	−5.5	−6.0	−5.6	−6.2	−6.2	−5.7
	TyrRS	−4.8	−5.0	−5.9	−5.1	−5.2	−4.8

**FIGURE 3:**
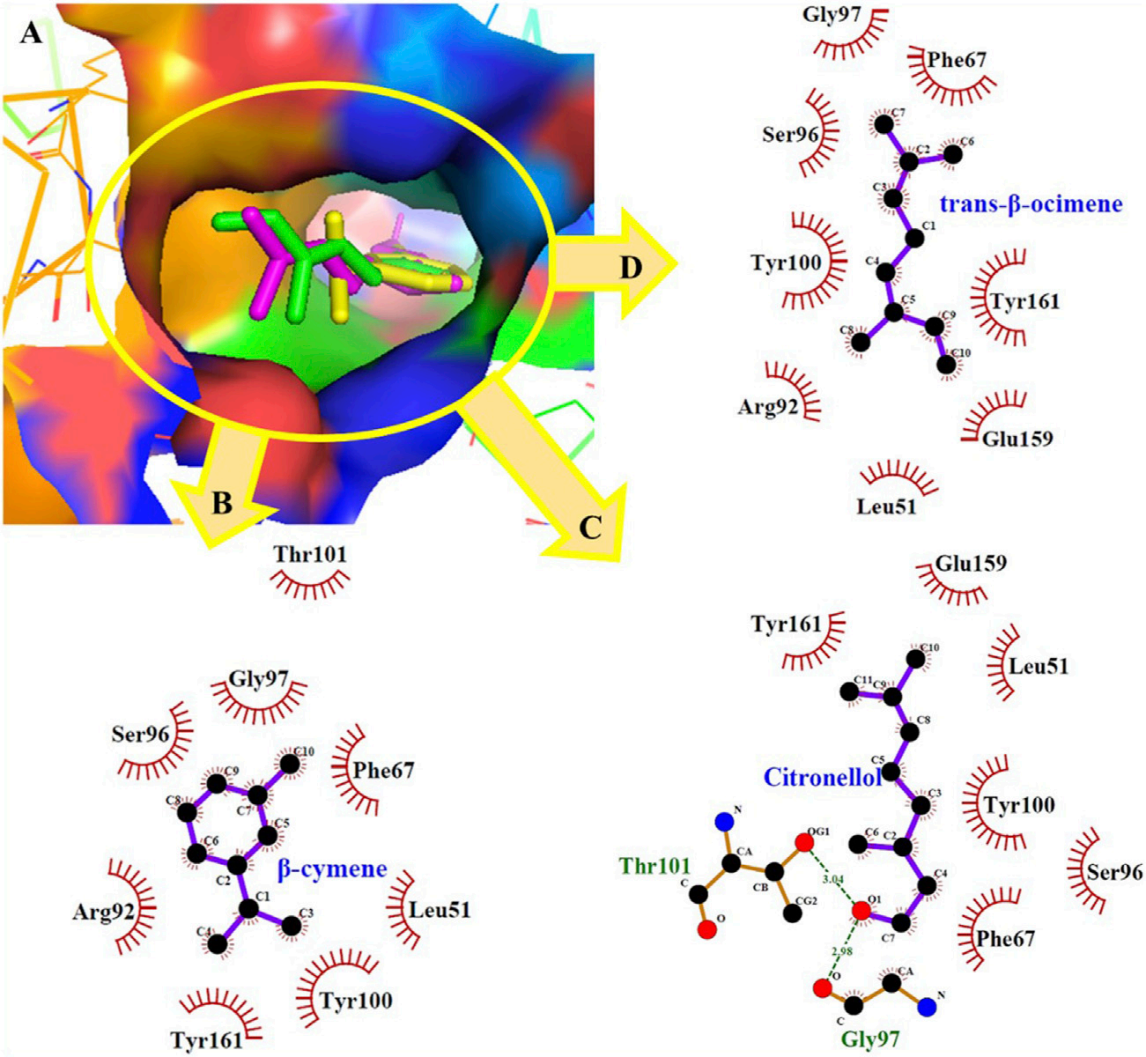
**(A)** Minimum energy conformation of docked *β*-cymene (yellow) *trans*-β-ocimene (green) and citronellol (magenta) in the binding pocket of thymidylate kinase of *Candida albicans*; **(B–D)** ligplot diagram.

In particular, among the docked compounds, citronellal showed the highest affinity for *Candida albicans* thymidylate kinase and *Herpes simplex* virus type-1 thymidine kinase, exhibiting minimum docking scores of −7.7 kcal/mol and −7.3 kcal/mol, respectively. In contrast, the maximal binding energy of the docked molecules was found to be in the range of −3.7 to −4.4 kcal/mol for 3C proteinase of the hepatitis A virus, justifying least affinity for this target. Nonpolar molecules like β-cymene and *trans*-β-ocimene participated only in short-range hydrophobic interactions, which have an indispensable value in the binding affinities among ligands and receptors. Although hydrophobic interactions are less restricted geometrically than hydrogen-bondings, they do contribute to specificity. dGMP (deoxyguanosine monophosphate) binds to the trimethoprim (TMP) binding site of *Candida albicans* thymidylate kinase with the help of non-bonding contributions from His64, Arg71, Thr101, and Tyr161 (Sinha and Rule, 2017). Similarly, docked compounds occupies the TMP binding area anchored by the Thr101, and Tyr161 residues (Figure 3). *Trans*-β-Ocimene and citronellol also exhibited appreciable binding affinities with this target adopting a similar conformation of TMP in the binding site.

### 3.6 ADME prediction

**FIGURE 4:**
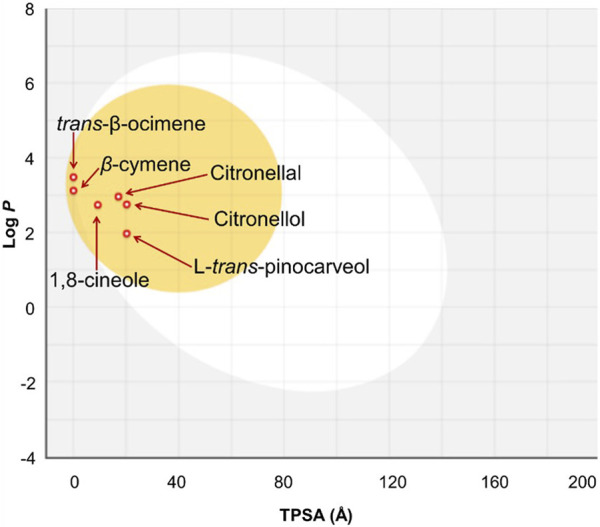
The boiled-egg graphs for the 1-8-cineole, *β*-cymene, citronellal, citronellol, *trans*-β-ocimene, and L-*trans*-pinocarveol using Swiss ADME web server.

The Swiss ADME program was manipulated for the anticipation of the ADME parameters of the 1-8-cineole, β-cymene, citronellal, citronellol, *trans*-*β*-ocimene, and L-*trans*-pinocarveol (DeLano, 2002; Daina and Zoete, 2016). All of the compounds were predicted to be appropriate drug candidates since none of them violated the Lipinski rule of five (Lipinski et al., 2012). As demonstrated in Figure 4, all investigated compounds had enough lipophilicity for enhanced gastrointestinal absorption and blood-brain barrier (BBB) crossing capacity. Gastrointestinal absorption and BBB permeability was predicted according to the white and yolk of the boiled-egg diagram, respectively. Table 8 summarizes the ADME prediction profile data. The bioavailability radar plots of each compound have been presented in Supplementary Figure S14 in the Supporting Material.

**TABLE 8:** Pharmacokinetic parameters computed by Swiss ADME web server.

Compounds	MW	RB	HBA	HBD	TPSA	Log*P*	GI absorption	BBB permeant	Lipinski violations
1-8-Cineole	154.25	0	1	0	9.23	2.74	High	Yes	0
*β*-Cymene	134.22	1	0	0	0	3.12	Low	Yes	1
Citronellal	154.25	5	1	0	17.07	2.96	High	Yes	0
Citronellol	156.27	5	1	1	20.23	2.75	High	Yes	0
*trans*-*β*-Ocimene	136.23	3	0	0	0	3.48	Low	Yes	0
L-*trans*-Pinocarveol	152.23	0	1	1	20.23	1.97	High	Yes	0

ADME: absorption, distribution, metabolism, excretion; BBB: blood-brain barrier; GI: gastrointestinal; HBA: hydrogen bond acceptor; HBD: hydrogen bond donor; MW: molecular weight; RB: rotatable bonds; TPSA: topological polar surface area.

## 4 Discussion

Genus Eucalyptus is known for its characteristic volatile oil content which accounts for its diverse medicinal and industrial applications. Despite comprising more than 800 species, there is sparsity in the literature concerning the volatile oil composition and the biological effectiveness of the different Eucalyptus species. The current study represents the first comparative GC-MS analysis of the volatile oil composition of three distinguished species of Eucalyptus growing in Egypt. The inflammatory modulating potentials and the antimicrobial activities of the essential oils of the three studied species were comparatively addressed. Eucalyptus-derived essential oils are known for their richness in terpenes and oxygenated derivatives. GC-MS investigations of the hydro-distilled oils obtained from the fresh leaves of the three species of Eucalyptus demonstrated the predominance of citronellal (ca 55%), β-cymene (ca. 12%), and trans-β-ocimene (ca 22%) in *E. citriodora*, *E. camaldulensis*, and *E. ficifolia*, respectively (Figure 1) with their concomitant absence or scarcity in the other oils, hence, they could be considered as marker compounds for their future identification.

The current study sheds the light on the influence of the habitat and the variations in the environmental conditions on altering the chemical compositions of *Eucalyptus species* essential oils. Despite the domination of terpenoids in the Egyptian *E. citriodora* oil (based on their relative area % in the GC-MS chromatogram as presented in Figure 1A) in the order of citronellal (54.9%) > citronellol (25.4%) > citronellol acetate (7.3%) > isopulegol (4.3%) > caryophyllene (2.8%), the essential oils collected from Benin (Sohounhloue et al., 1996) and Thailand (Chahomchuen et al., 2020) showed notable qualitative and quantitative variations in their GC-MS chromatographic profiles with an order of citronellal (65.4%) > citronellol (13.0%) > isopulegol (10.3%) > citronellyl acetate (2.0%) dominating the former and citronellal (68.4%) > citronellol (9.5%) > cyclohexanol (6.4%) > γ-terpinene (1.3%) dominating the later oil.

The essential oil of *E. camaldulensis* displayed predominance in β-cymene and 1,8-cineole (Eucalyptol) constituting ca. 12.77% and 11.74%, respectively, followed by β-pinene (9.86%) and spathulenol (9.05%) which constituted almost the same percentage. *E. camaldulensis* oils obtained from Thailand (Chahomchuen et al., 2020) and Morocco (Ez-Zriouli et al., 2023) demonstrated major quantitative and qualitative variations in their GC-MS chromatographic profiles compared to the Egyptian variety with 1,8-cineole being the predominant terpene representing ca. 31% of the total chromatographic area, followed by γ-terpinene (22%), α-phellandrene (21%), o-cymene (5.7%), α-pinene (3%), and α-terpinolene (2%) in the Thai-derived *E. camaldulensis* oil while p-cymene (35.11%), ɣ-eudesmol (11.9%), L-linalool (11.51%), and piperitone (10.28%) predominating the Moroccan-derived *E. camaldulensis* oil.The Malaysian *E. camaldulensis* oil showed a predominance of γ-terpinene (71%), o-cymene (17.6%), and terpinen-4-ol (7%) (Mubarak et al., 2014). These variations were even recognizable between Eucalyptus oils collected from different regions within the same land. Previous study on *E. camaldulensis* collected in Egypt, yet from another city (Alexandria) (Mohamed and Abdelgaleil, 2008), showed totally different metabolites with 1,8-cineole (45.4%) (−)-spathulenol (32.3%), and bicyclogermacrene (11.2%) being the major metabolites. Moreover, variations were also prominent in the chemical profiles of Eucalyptus essential oils collected at different seasons. It is noteworthy to hint that previous GC-MS analysis of an Egyptian *E. citriodora* oil collected in the winter season showed an entirely different chromatographic profile with 3-hexen-1-ol (31.2%) being the major metabolite followed by *cis*-geraniol (19.6%), citronellol acetate (13.6%), and 5-hepten-1-ol, 3,6-dimethyl (13.1%) (Abd El-Mageed et al., 2011). In the current study, where *E. citriodora* oil was collected in summer, citronellal (54.9%) was the major constituent however in the wintertime it represented only 9.3% of the total oil content.

To the best of our knowledge, this is the first report on the GC-MS analysis of the oil of *E. ficifolia* growing in Egypt. The essential oil of *E. ficifolia* showed predominance of trans-β-ocimene (22.40%) followed by 1,8-cineole (Eucalyptol) (13.51%) and L-trans-pinocarveol (12.55%) which showed almost comparable percentages. The total monoterpene hydrocarbons and monoterpenoids in the current study represented 71.7% of *E. ficifolia* oil, however, that one collected from Tunisia (Elaissi et al., 2010) showed richness in oxygenated sesquiterpenes like (*E*,*E*)-farnesol, which was identified as the major volatile constituent.

*In-vitro* studies investigating the inflammatory-modulating effects of the studied oils revealed a superior inhibitory activity of *E. citriodora* oil (32% inhibition) on the release of superoxide anion radical compared to *E. ficifolia* (19.3% inhibition) and *E. camaldulensis* (13.6% inhibition) suggesting a better anti-inflammatory activity for *E. citriodora* over the other two species (Table 2). This prominent activity of *E. citriodora* oil is probably associated with its citronellal content, which has previously been reported for its potent anti-inflammatory and antioxidant capability (Melo et al., 2011; Quintans-Júnior et al., 2011). Similarly, the current docking study revealed good docking energy of citronellal to human neutrophil elastase (−5.33 kcal/mol) comparable to the previous study reporting a binding energy of −3.92 kcal/mol to the same enzyme (Sivamani et al., 2012).

Neutrophil elastase is part of the innate host defense against microbes by promoting inflammation, facilitating leukocyte transmigration, degrading bacterial virulence elements, initiating inflammatory cascade, beside its bactericidal activities specially against Gram-negative bacteria. The essential oil of *E. citriodora* displayed the strongest *in vitro* inhibition to human elastase (31.8% inhibition) followed by *E. camaldulensis* essential oil which demonstrated 21.3% suppression of human elastase. On the contrary, *E. ficifolia* oil displayed an enhancing effect on elastase by increasing (rather than inhibiting) elastase levels, thus boosting the immune system to better fight against the microbial infection (Table 3). These results suggested that *E. citriodora* and *E. camaldulensis* oils probably display better anti-inflammatory activities than *E. ficifolia*, yet less bactericidal activities (Table 6). Results of the antibacterial activities of the investigated Eucalyptus oils demonstrated the superior inhibitory activity of *E. ficifolia* oil against all the studied bacterial strains. Results agree with the previous study reporting the inhibitory efficacy of 1,8-cineole and terpinen-4-ol against *Escherichia coli* and *Staphylococcus aureus* (Moghimi et al., 2017). 1,8-Cineole and terpinen-4-ol represented 13.51% and 6.91% of *E. ficifolia* oil in the current study compared with 11.74% and 2.27% in *E. camaldulensis* (Carson and Riley, 1995; Vuuren and Viljoen, 2007), however they were almost absent in the oil of *E. citriodora*. This might explain the best activity observed for *E. ficifolia* oil against the tested bacteria. *E. camaldulensis* oil has been previously reported to display antibacterial effect against *S. aureus* and *E. coli* resistant strains due to the synergistic activity of its components (Chaves et al., 2018; Aleksic Sabo and Knezevic, 2019) which is in agreement with the results described in the current study.

*E. ficifolia* oil exhibited likewise the strongest antiviral activity against hepatitis A, *Herpes simplex* and coxsackie viruses with the least IC_50_ compared to the other studied Eucalyptus species and comparable to the standard acyclovir drug (Table 4). This potent antiviral activity of *E. ficifolia* oil might be attributed to its enhanced effect on the release of elastase by human neutrophils, hence increasing the anti-pathogenic inflammatory response of neutrophils, which correlates well with the observed antiviral and antibacterial activities and provides further support for its potential utility as an anti-infective agent.

Meanwhile, *E. camaldulensis* essential oil had demonstrated the strongest antifungal activity against *Candida albicans* among the studied oils. It coincides with the previous reported literature of its antifungal activity (Dogan et al., 2017) in addition to the reported fungistatic activity of its major constituents, β-cymene and β-pinene, (Tampieri et al., 2005; Höferl et al., 2009). Molecular docking investigations were employed to identify a protein target that could potentially serve as a plausible mechanism for establishing a correlation between the observed *in vitro* anti-inflammatory, antiviral, antibacterial, and antifungal activity and the respective phytoconstituents. The targeted protein related to inflammation was human neutrophil elastase, whereas viral proteins consisted of 3C proteinase of hepatitis A virus, and DNA polymerase and thymidine kinase of *Herpes simplex* virus type-1. Dihydrofolate reductase, DNA gyrase, pantothenate synthetase, and tyrosyl-tRNA synthetase targets were selected from *Staphylococcus aureus* bacteria. Proteins associated with *Candida albicans* include CYP51, dihydrofolate reductase, *N*-myristoyltransferase, secreted aspartic proteinase, and thymidylate kinase.

A docking score or binding energy value is used to measure ligand-receptor affinity in molecular docking. A higher affinity usually corresponds to greater biological activity. Among the docked targets, maximum affinity was noted with the thymidylate kinase of *Candida albicans* depicting the highest affinity for β-cymene, *trans*-β-ocimene, citronellol, and citronellal with binding energies of −7.7, 7.0, −6.6, and −6.4 kcal/mol, respectively. However, CYP51 was observed as the second most preferred target from the same organism, displaying −6.8 and −6.7 kcal/mol by L-*trans*-pinocarveol and 1,8-cineole, respectively.

The *in silico* molecular docking experiments were accomplished for better understanding of the affinity of the major components of Eucalyptus essential oils towards the different pharmacological targets related to the evaluated inflammatory, antibacterial, antiviral, and antifungal activities. Results demonstrated the minimum binding energies of citronellal, the major volatile constituent of *E camaldulensis* against all the targets related to *Candida albicans* including CYP51, dihydrofolate reductase, N-myristoyltransferase, secreted aspartic proteinase and thymidylate kinase. This justifies the observed potent *in vitro* antifungal activity of *E. camaldulensis oil.* Moreover, the low binding energies demonstrated by citronellal (−5.3) and 1,8-cineole (−4.9) against elastase target explained the strong inhibitory activities of *E. citriodora* and *E. camaldulensis* oils respectively. The potent antibacterial and antiviral activities of *E. ficifolia* is probably due to the synergetic activity of its major constituents, 1,8-cineole, *trans*-β-ocimene and L-*trans*-pinocarveol. This can be explained from the marked low binding energies of the later constituents against the tested targets especially 3C proteinase, dihydrofolate reductase and pantothenate synthetase. The ADME prediction studies suggested the promising bioavailability of the essential oils’ major constituents demonstrating enough lipophilicity for passing the BBB and have adequate GI absorption.

## 5 Conclusion

Substantial variation has been witnessed among the chemical composition of the hydrodistilled essential oils of *Eucalyptus citriodora*, *Eucalyptus camaldulensis*, and *Eucalyptus ficifolia* leaves, growing in Egypt. These differences in chemical composition resulted in comprehensive variabilities in their inflammatory modulating effects as well as their antibacterial, antiviral, and anti-fungal activities. *In silico* studies of the major volatile constituents supported the demonstrated bioactivity. The essential oils of *E. citriodora* and *E. ficifolia* proved to be promising candidates for incorporation into anti-inflammatory and antimicrobial pharmaceutical products, respectively. Further *in vivo* and clinical studies are recommended to validate the safety and efficacy of their incorporation.

## Data Availability

The original contributions presented in the study are included in the article/Supplementary Material, further inquiries can be directed to the corresponding authors.
