# Supplementary material for: Eucalyptus-derived essential oils alleviate microbes and modulate inflammation by suppressing superoxide and elastase release
Source: Front Pharmacol. 2023 Nov 21;14:1218315. doi: 10.3389/fphar.2023.1218315 (PMC10703155; doi:10.3389/fphar.2023.1218315)
Supplement: Supplementary file 1 [file Table1.DOCX]

Supplementary Material

Eucalyptus-derived Essential Oils Alleviate Microbes and Modulate Inflammation by Suppressing Superoxide and Elastase Release

Shaimaa Fayez^1†^_,_ Mariam I. Gamal El-Din^1†^, Saad A. Moghannem^2^, Faizul Azam^3^, Mohamed El-Shazly^1*^, Michal Korinek^4,5^, Yu-Li Chen^6,7^, Tsong-Long Hwang^5,6,7,8*^, Nouran M. Fahmy^1^

*** Correspondence:**Prof. Mohamed elshazly
[mohamed.elshazly@pharma.asu.edu.eg](mailto:mohamed.elshazly@pharma.asu.edu.eg)

Prof. Tsong-Long Hwang

[htl@mail.cgu.edu.tw](mailto:htl@mail.cgu.edu.tw)

**^†^**These authors contributed equally to this work

# Supplementary Figures


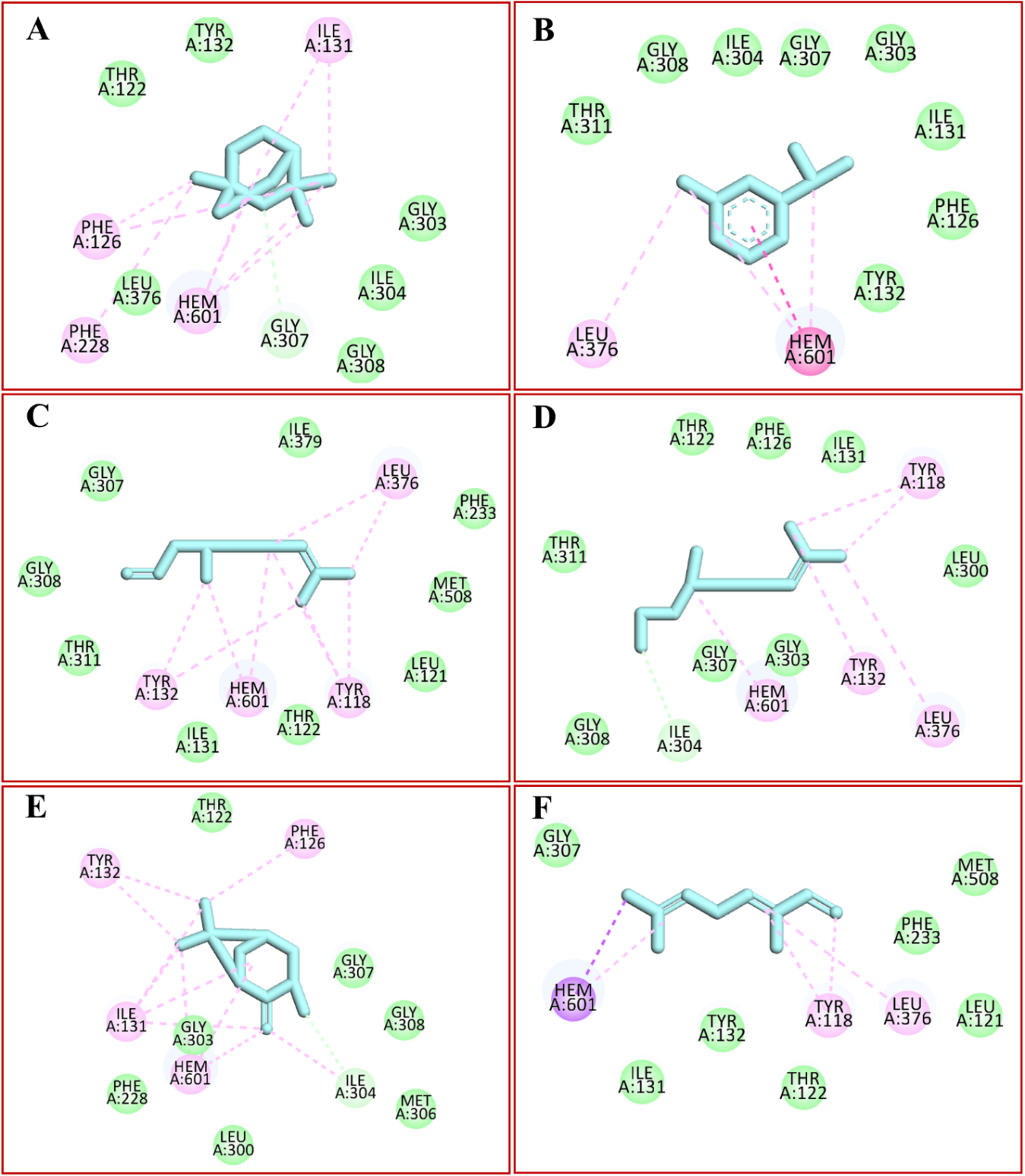


**Supplementary Figure 1.** 2D depiction of the intermolecular interactions between docked compounds 1-8-cineole (A), *β*-cymene (B), citronellal (C), citronellol (D), *trans*-beta-ocimene (E), and L-*trans*-pinocarveol (F) and CYP51 of *Candida albicans*.


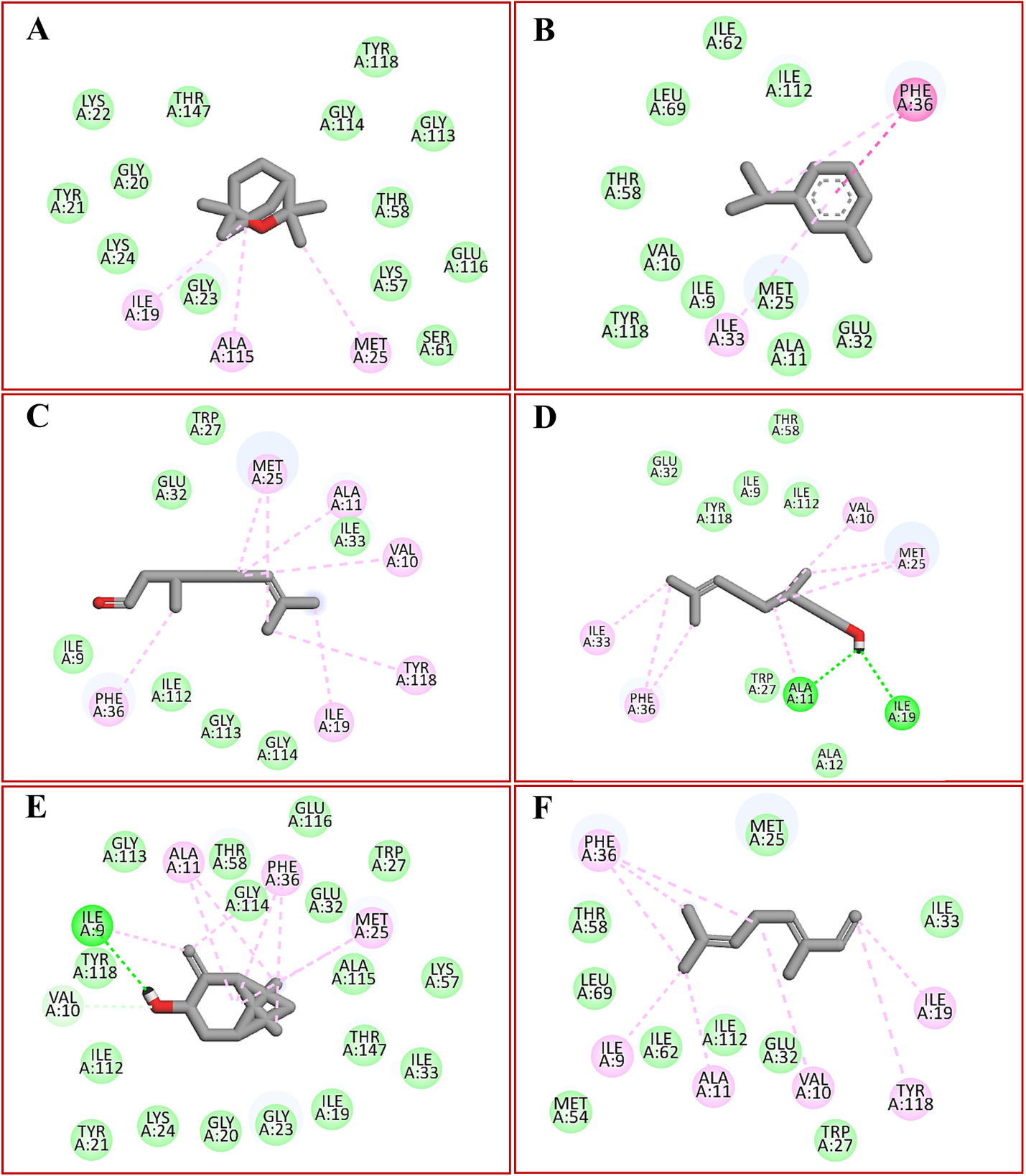


**Supplementary Figure 2.** 2D depiction of the intermolecular interactions between docked compounds 1-8-cineole (A), *β*-cymene (B), citronellal (C), citronellol (D), *trans*-beta-ocimene (E), and L-*trans*-pinocarveol (F) and dihydrofolate reductase of *Candida albicans*.


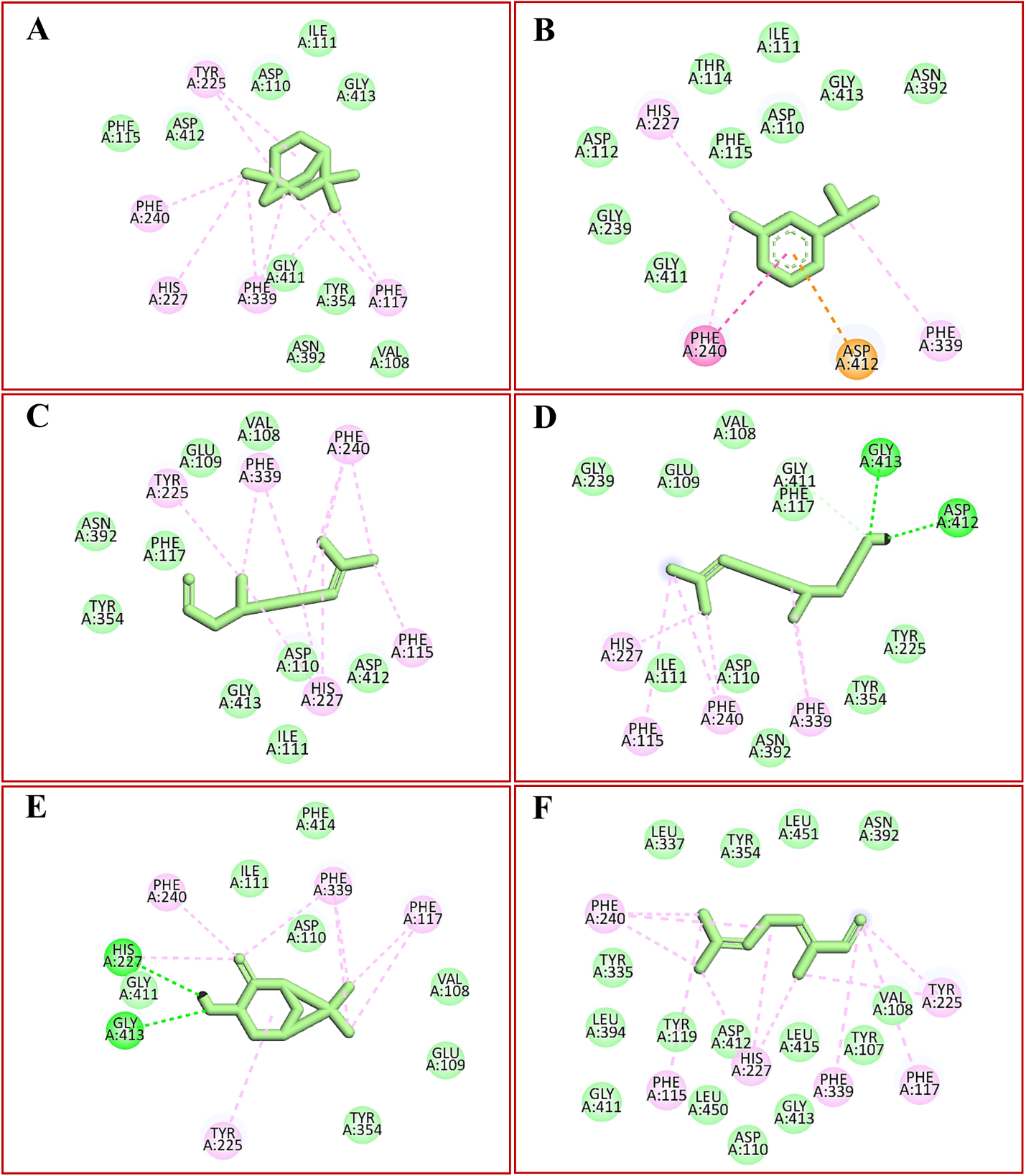


**Supplementary Figure 3. 2**D depiction of the intermolecular interactions between docked compounds 1-8-cineole (A), *β*-cymene (B), citronellal (C), citronellol (D), *trans*-beta-ocimene (E), and L-*trans*-pinocarveol (F) and *N*-myristoyl transferase of *Candida albicans*.


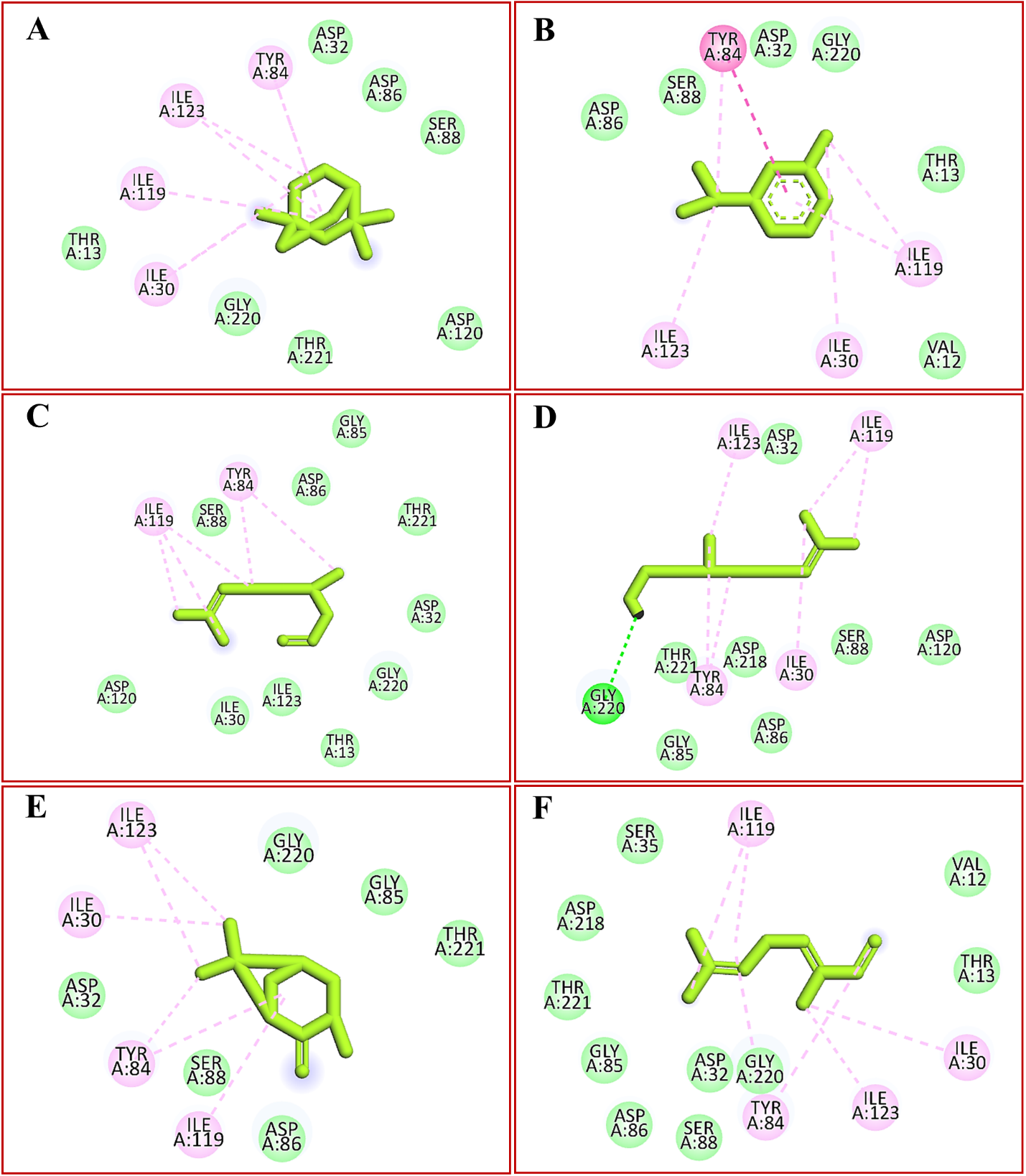


**Supplementary Figure 4.** 2D depiction of the intermolecular interactions between docked compounds 1-8-cineole (A), *β*-cymene (B), citronellal (C), citronellol (D), *trans*-beta-ocimene (E), and L-*trans*-pinocarveol (F) and secreted aspartic proteinase of *Candida albicans*.


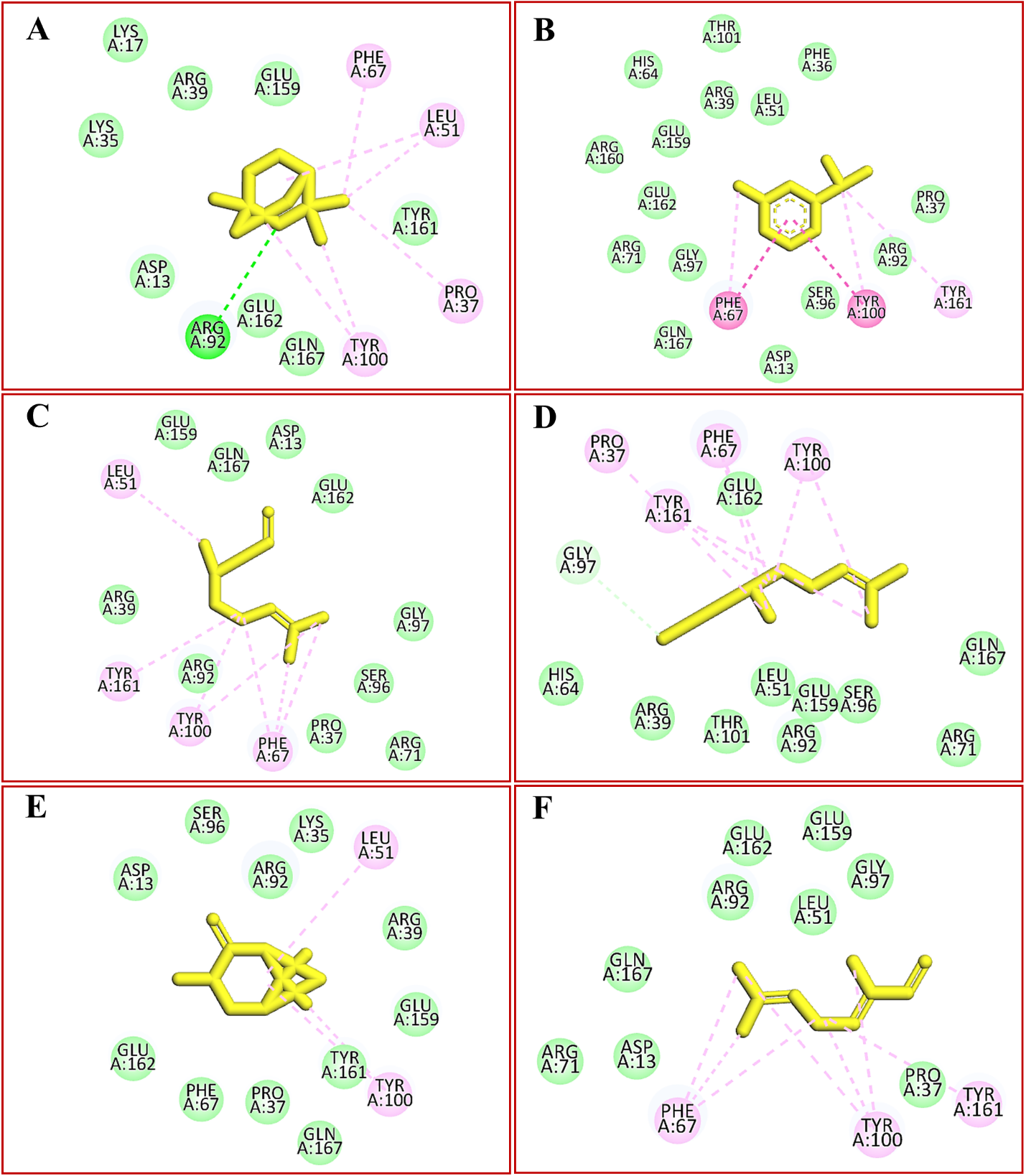


**Supplementary Figure 5.** 2D depiction of the intermolecular interactions between docked compounds 1-8-cineole (A), *β*-cymene (B), citronellal (C), citronellol (D), *trans*-beta-ocimene (E), and L-*trans*-pinocarveol (F) and thymidylate kinase of *Candida albicans*.


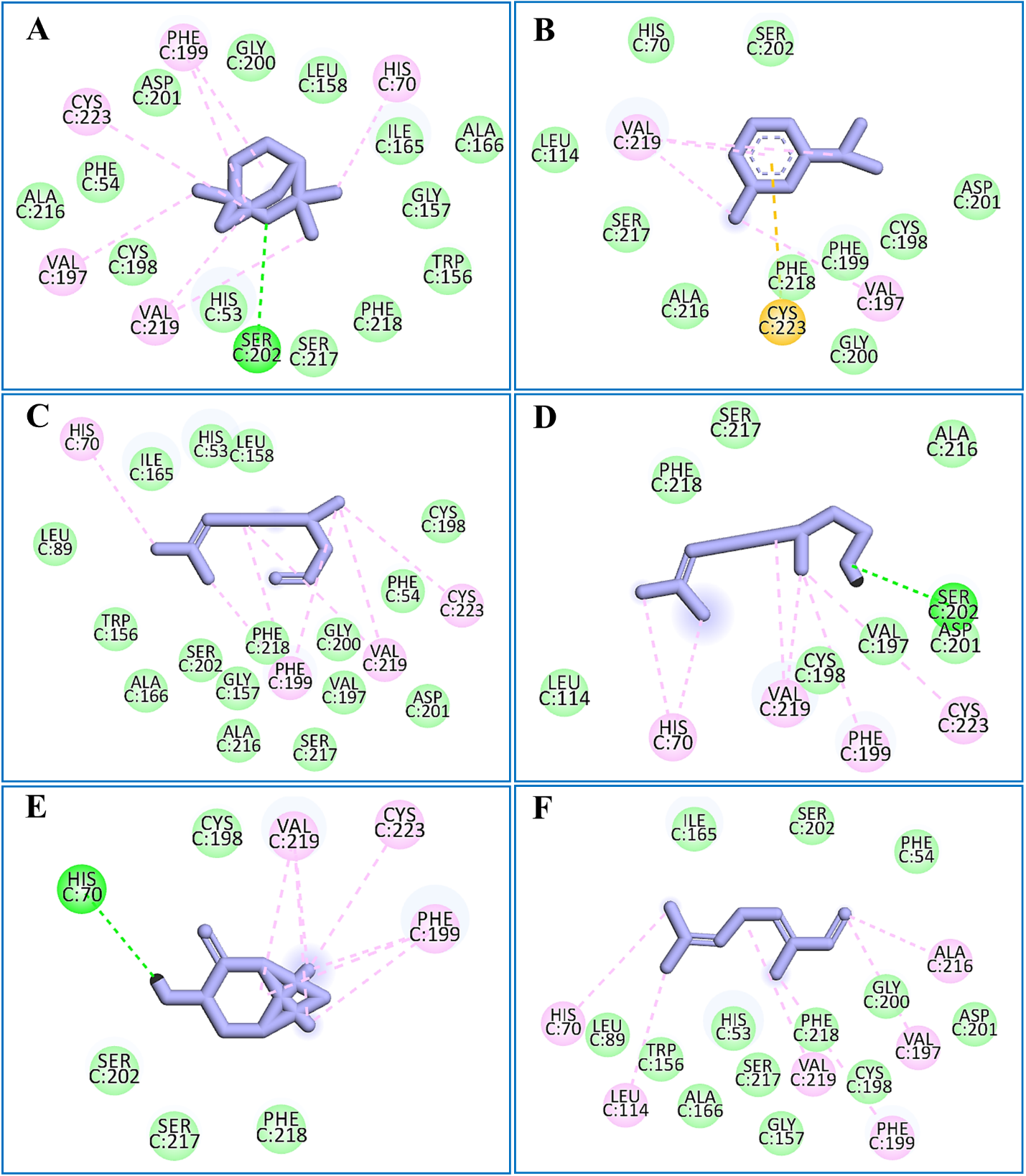


**Supplementary Figure 6.** 2D depiction of the intermolecular interactions between docked compounds 1-8-cineole (A), *β*-cymene (B), citronellal (C), citronellol (D), *trans*-beta-ocimene (E), and L-*trans*-pinocarveol (F) and human neutrophil elastase.


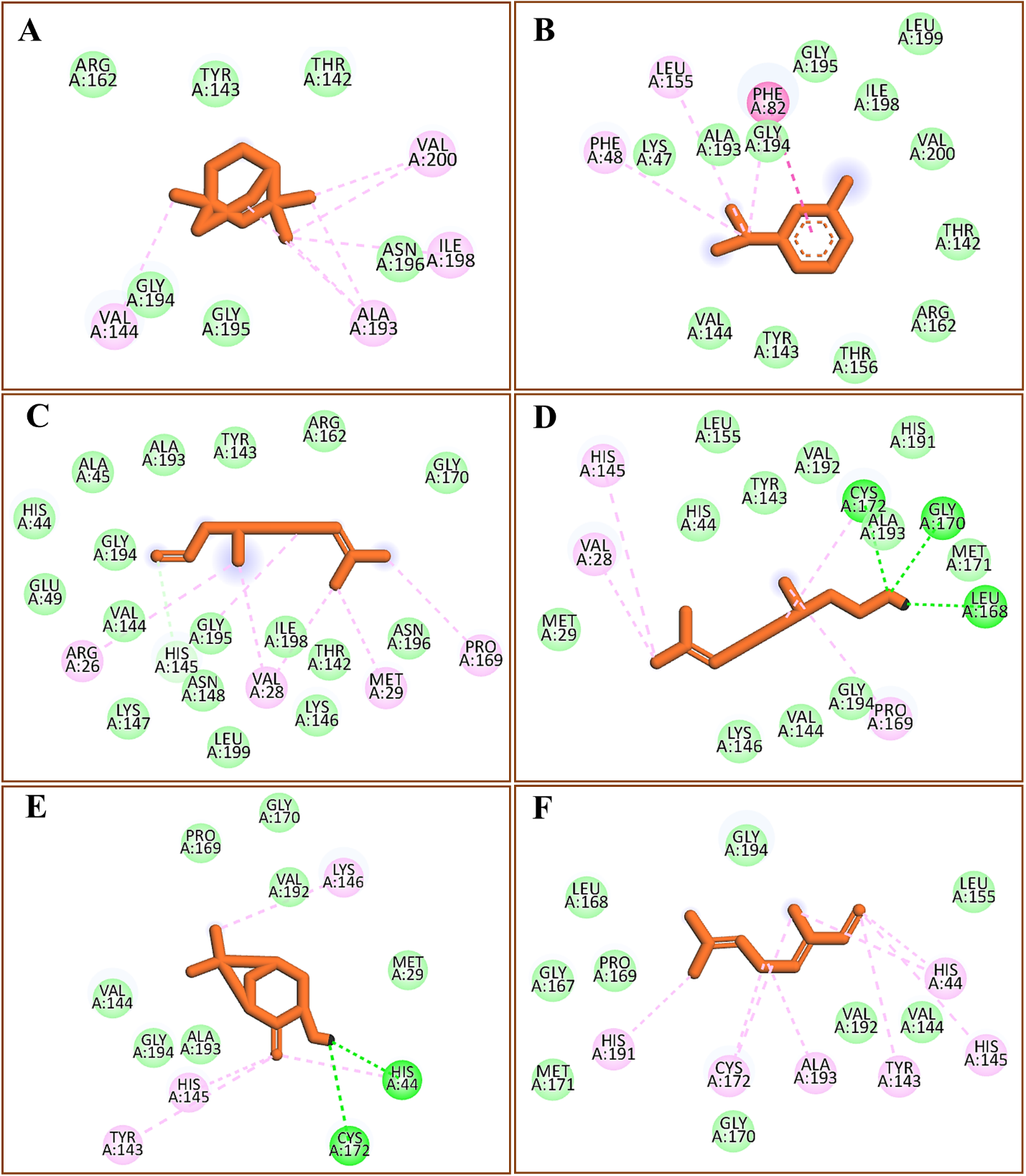


**Supplementary Figure 7.** 2D depiction of the intermolecular interactions between docked compounds 1-8-cineole (A), *β*-cymene (B), citronellal (C), citronellol (D), *trans*-beta-ocimene (E), and L-*trans*-pinocarveol (F) and 3C proteinase of hepatitis A virus.


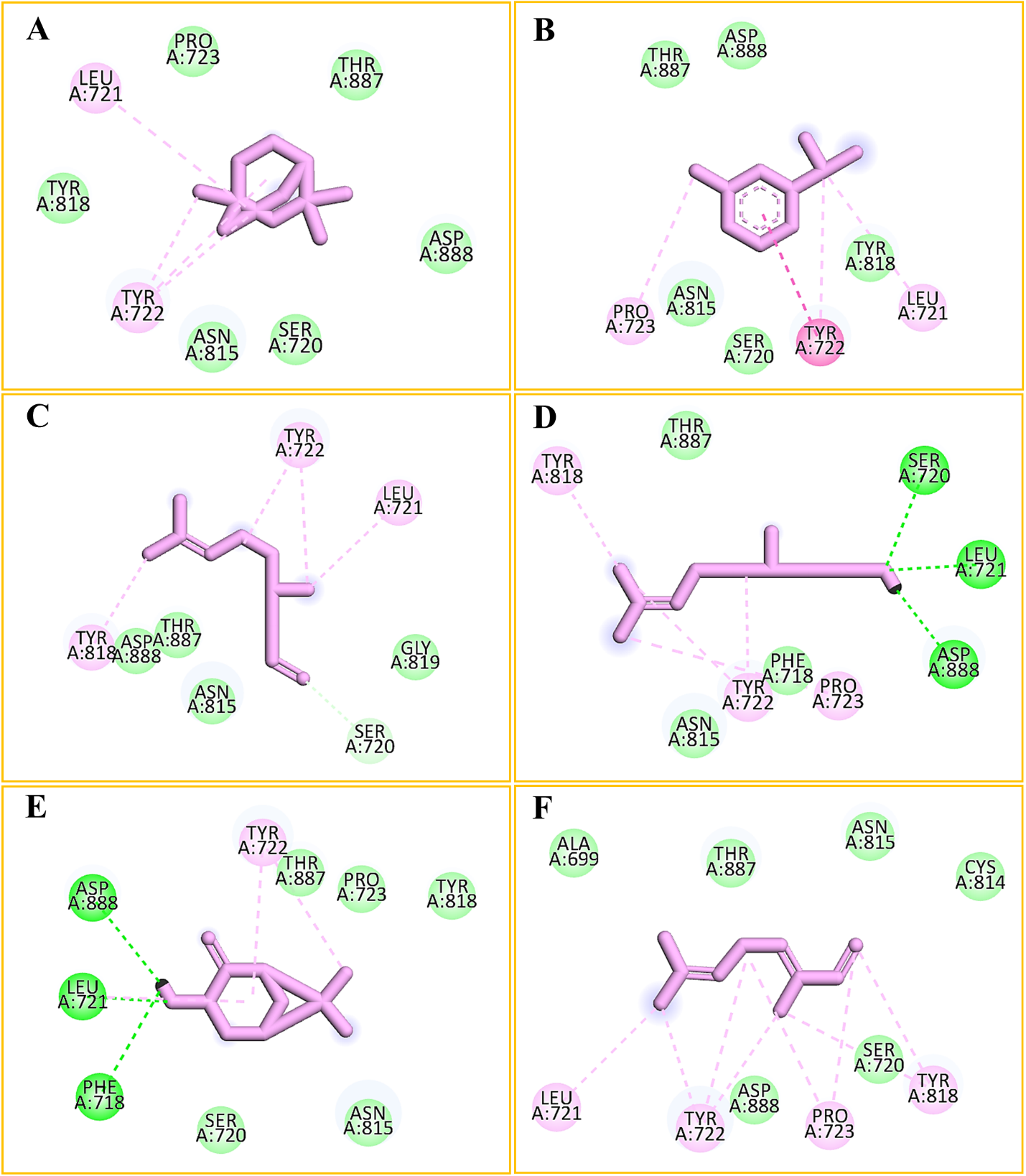


**Supplementary Figure 8.** 2D depiction of the intermolecular interactions between docked compounds 1-8-cineole (A), *β*-cymene (B), citronellal (C), citronellol (D), *trans*-beta-ocimene (E), and L-*trans*-pinocarveol (F) and DNA polymerase of *Herpes simplex* virus type-1.


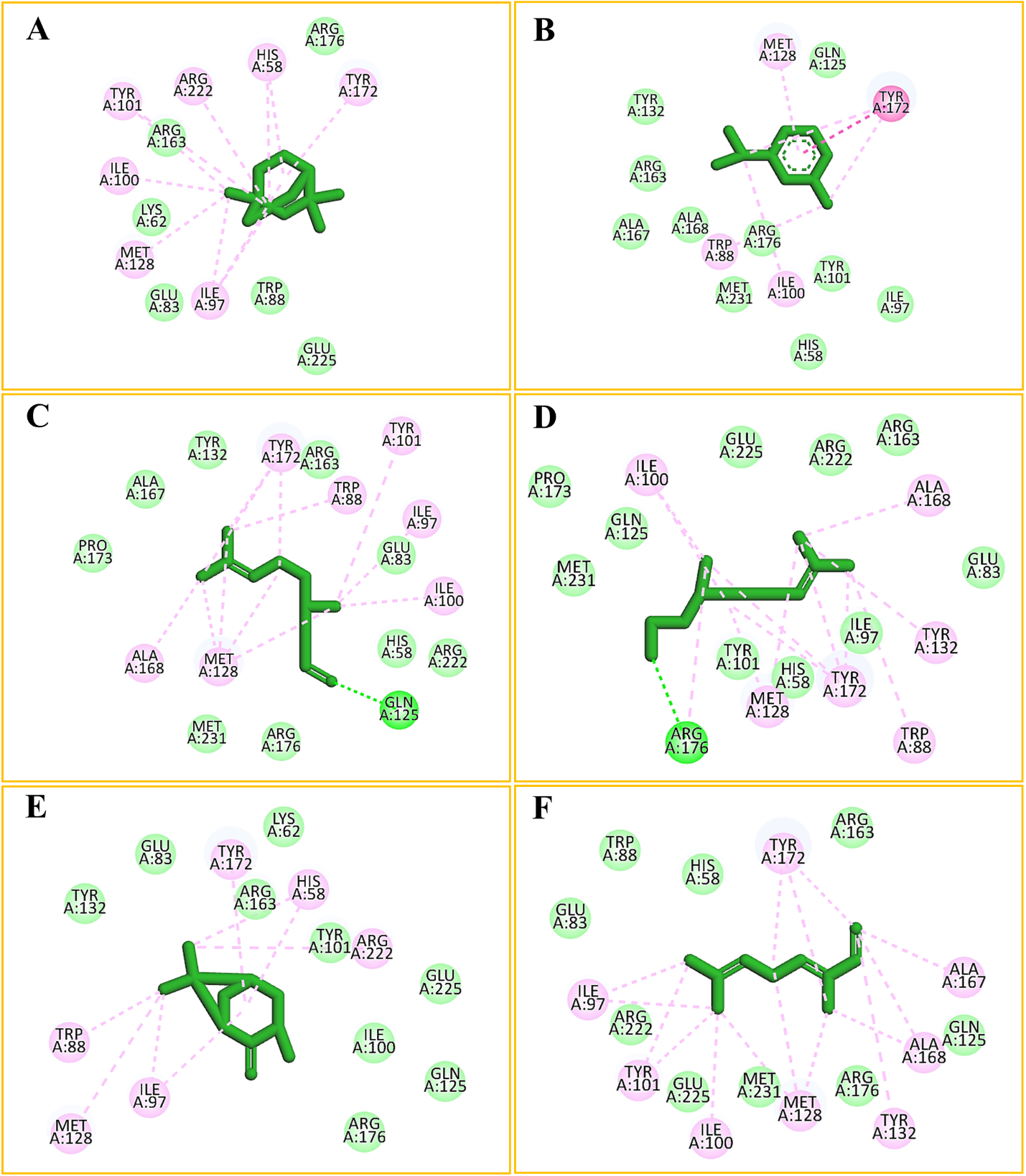


**Supplementary Figure 9.** 2D depiction of the intermolecular interactions between docked compounds 1-8-cineole (A), *β*-cymene (B), citronellal (C), citronellol (D), *trans*-beta-ocimene (E), and L-*trans*-pinocarveol (F) and thymidine kinase of *Herpes simplex* virus type-1.


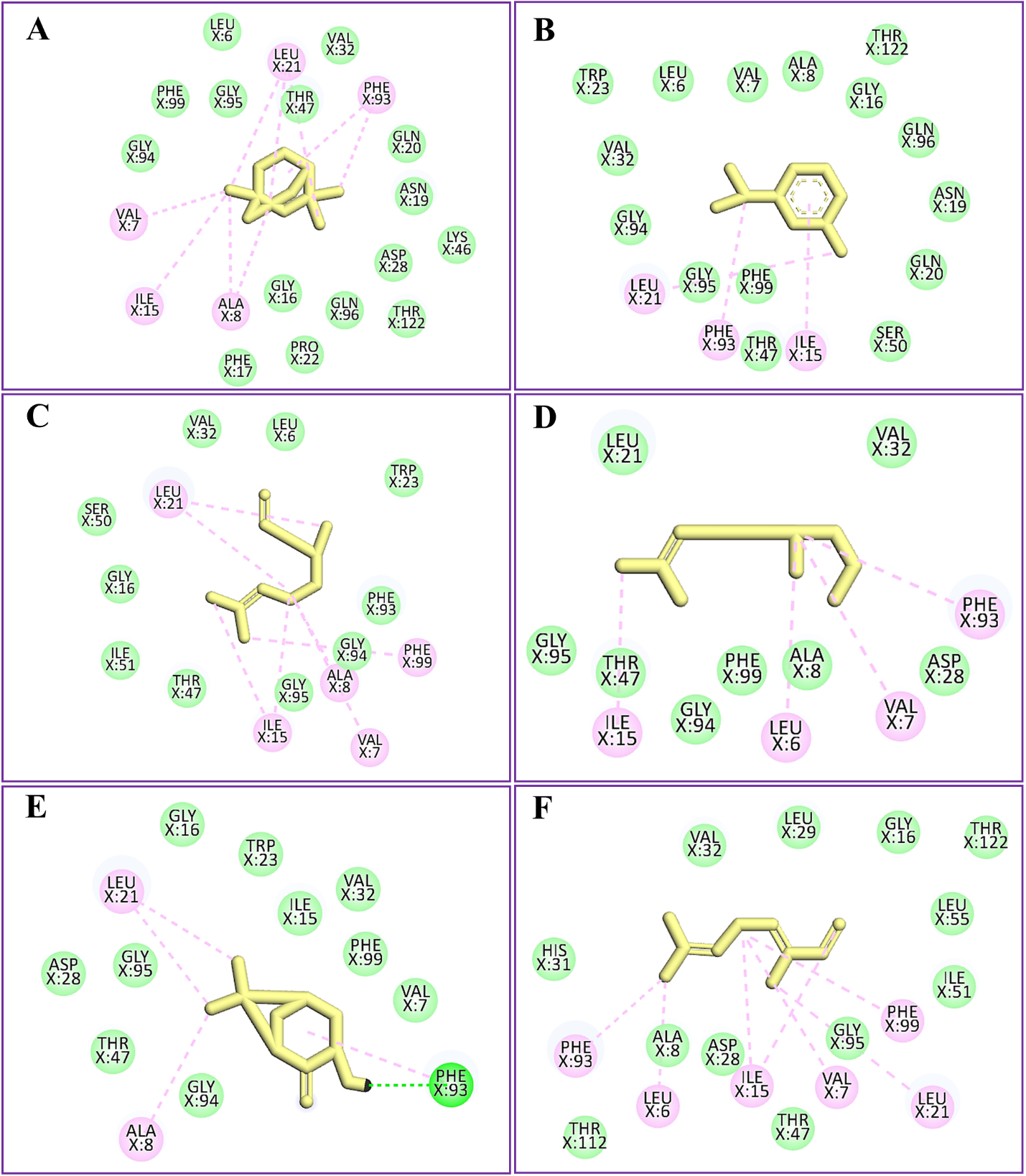


**Supplementary Figure 10.** 2D depiction of the intermolecular interactions between docked compounds 1-8-cineole (A), *β*-cymene (B), citronellal (C), citronellol (D), *trans*-beta-ocimene (E), and L-*trans*-pinocarveol (F) and dihydrofolate reductase of *Staphylococcus aureus*.


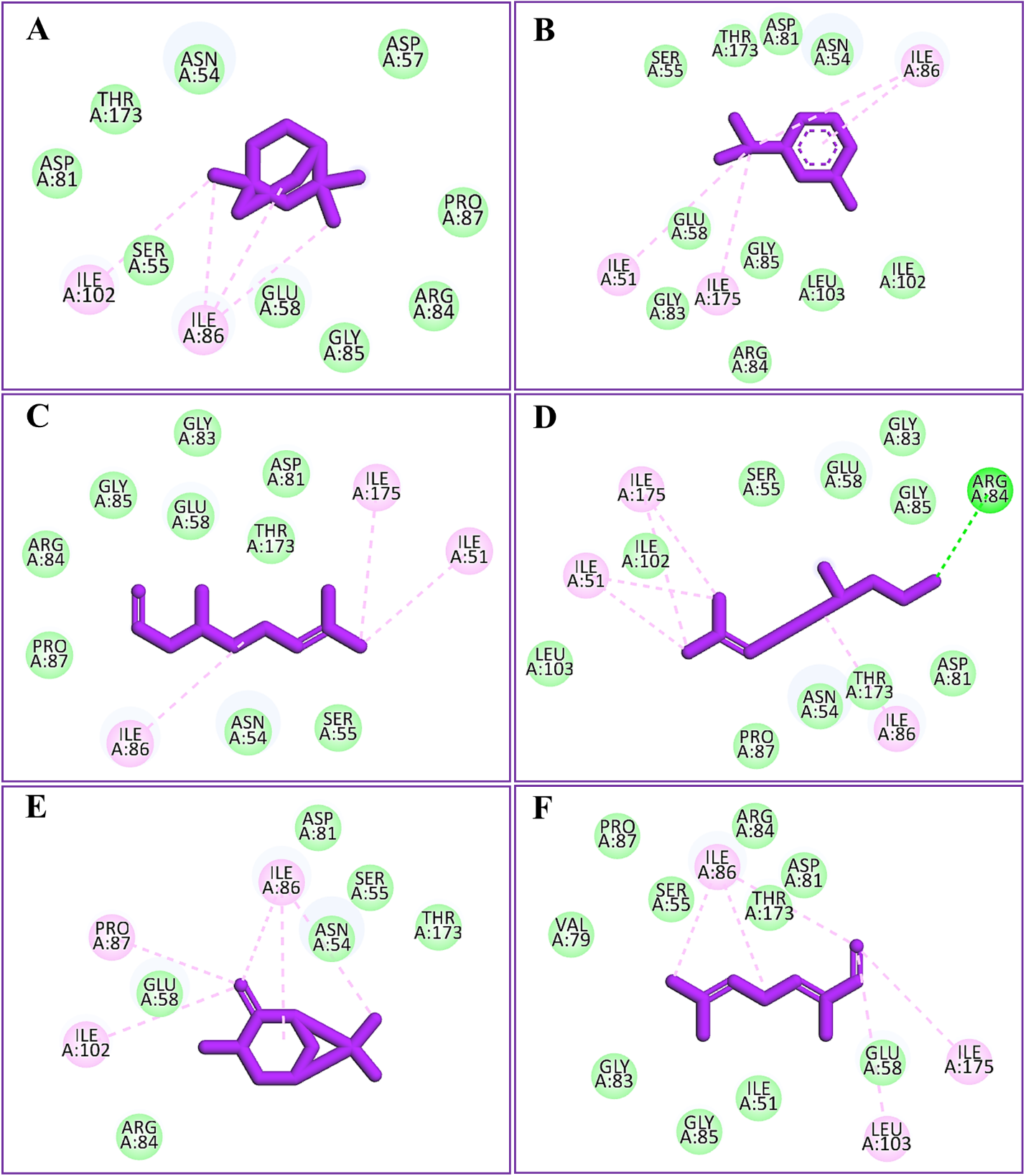


**Supplementary Figure 11.** 2D depiction of the intermolecular interactions between docked compounds 1-8-cineole (A), *β*-cymene (B), citronellal (C), citronellol (D), *trans*-beta-ocimene (E), and L-*trans*-pinocarveol (F) and DNA gyrase of *Staphylococcus aureus*.


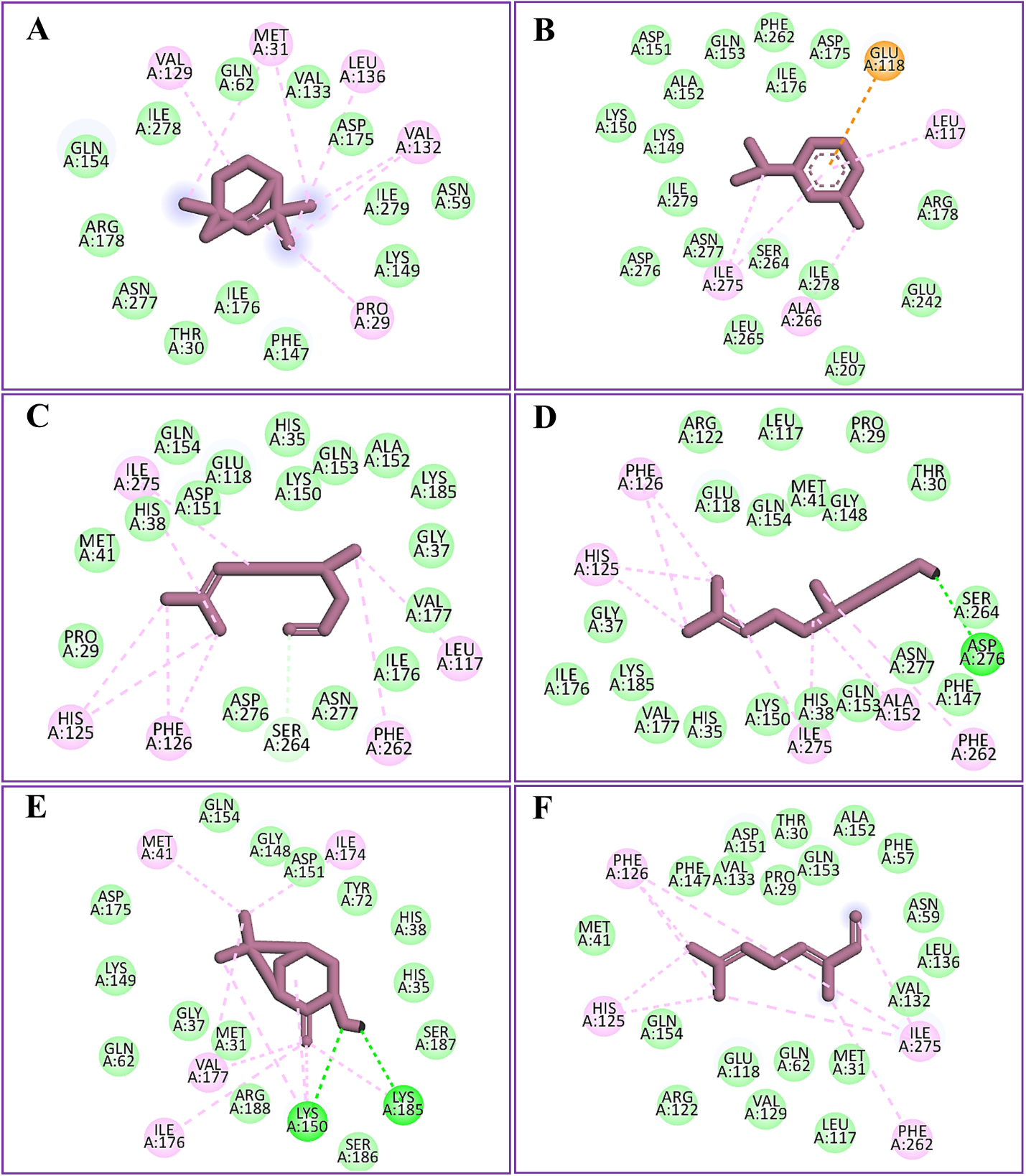


**Supplementary Figure 12.** 2D depiction of the intermolecular interactions between docked compounds 1-8-cineole (A), *β*-cymene (B), citronellal (C), citronellol (D), *trans*-beta-ocimene (E), and L-*trans*-pinocarveol (F) and pantothenate synthetase of *Staphylococcus aureus*.


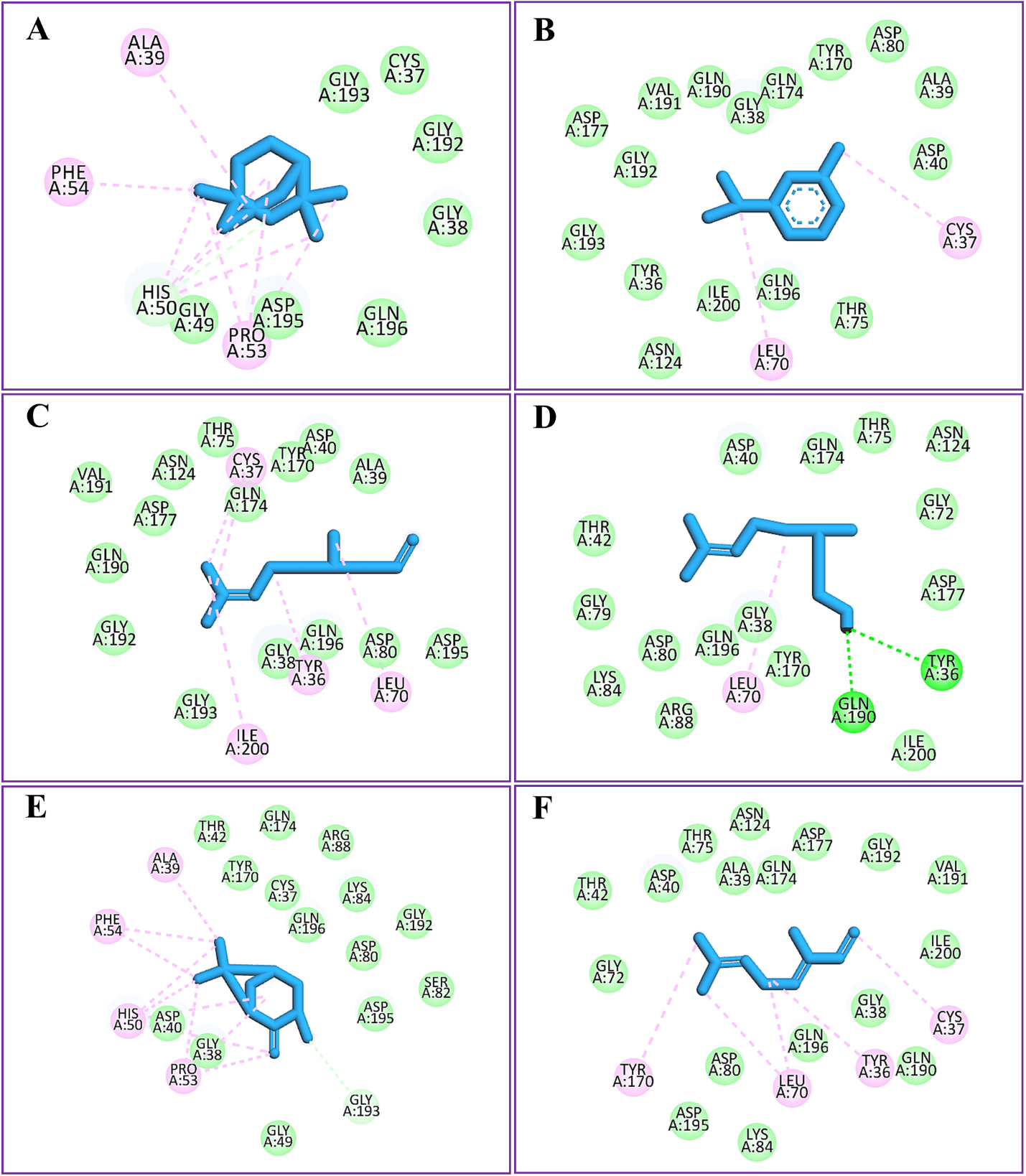


**Supplementary Figure 13.** 2D depiction of the intermolecular interactions between docked compounds 1-8-cineole (A), *β*-cymene (B), citronellal (C), citronellol (D), *trans*-beta-ocimene (E), and L-*trans*-pinocarveol (F) and tyrosyl-tRNA synthetase (TyrRS) of *Staphylococcus aureus*.


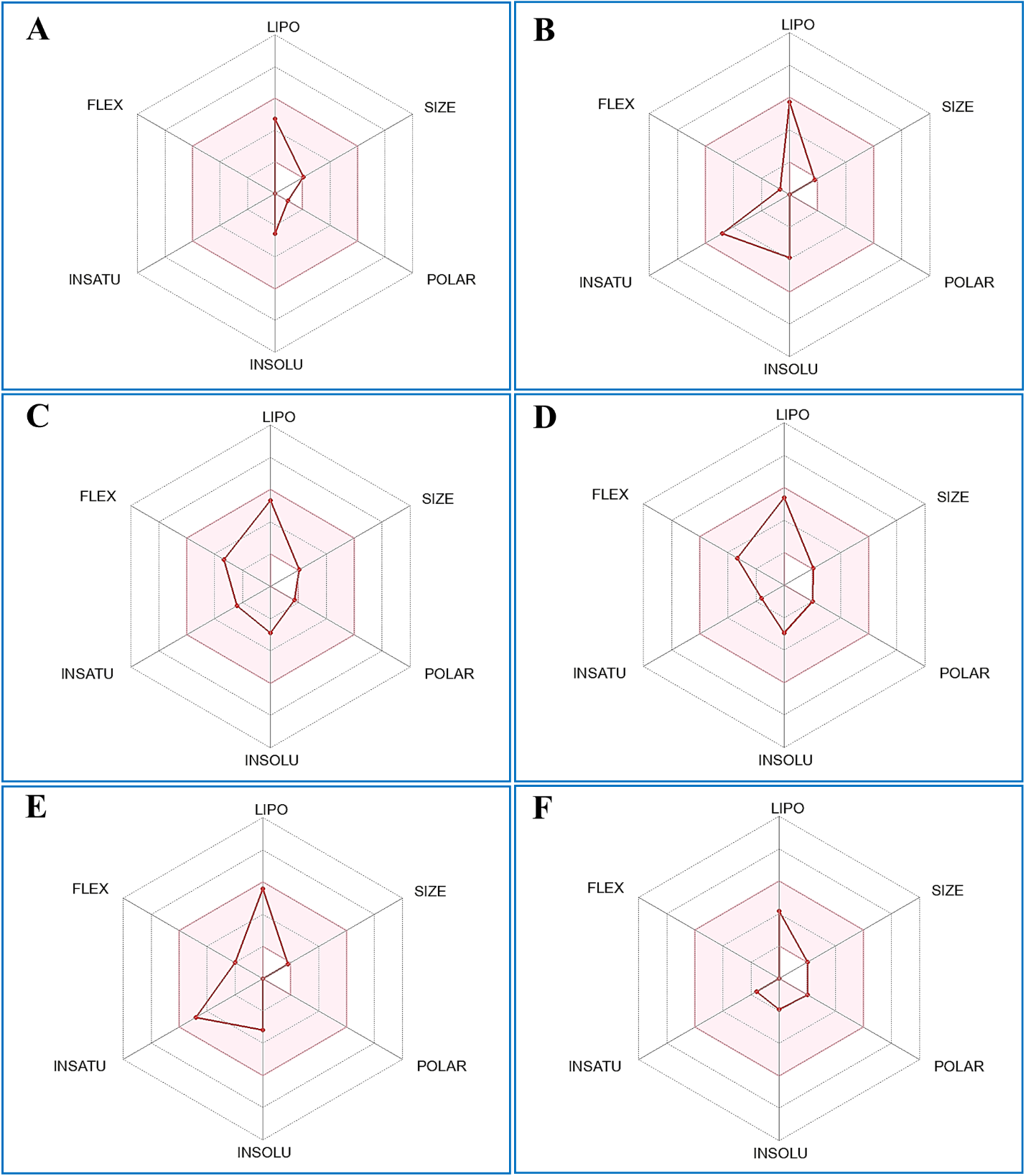


**Supplementary Figure 14.** The bioavailability radar plots of the 1-8-cineole (A), *β*-cymene (B), citronellal (C), citronellol (D), *trans*-*β*-ocimene (E), and L-*trans*-pinocarveol (F).
